# Cross-species transcriptomics identify mineralocorticoid receptor pathway overactivation as a central driver of ocular rosacea

**DOI:** 10.1038/s41467-026-71945-4

**Published:** 2026-04-16

**Authors:** Linxin Zhu, Nilufer Yesilirmak, Daniela Rodrigues-Braz, Emmanuelle Gélizé, Coralie Lheure, Xavier Morel, Jean-Louis Bourges, Frédéric Jaisser, Min ZHAO, Francine Behar-Cohen

**Affiliations:** 1 Centre de Recherche des Cordeliers, INSERM, Université Paris Cité, Sorbonne Université, UMRS 1138, Team «Physiopathology of Ocular Diseases: Therapeutic Innovations», Paris, France; 2 Ophtalmopole, Assistance Publique -Hôpitaux de Paris (AP-HP), Cochin Hospital, Paris, France; 3 Department of Ophthalmology, Ankara Yildirim Beyazit University, Ankara City Hospital, Ankara, Turkey; 4 Department of Dermatology, AP-HP, Cochin Hospital, Paris, France; 5 INSERM, UMRS 1166, Hôpital La Pitié Salpétrière, Sorbonne Université, Paris, France; 6 Université de Lorraine, INSERM Centre d’Investigations Cliniques-Plurithématique 1433, UMR 1116, CHRU de Nancy, French-Clinical Research Infrastructure Network (F-CRIN) INI-CRCT, Nancy, France; 7 Hopital Foch, Department of Ophthalmology, Suresnes, France

**Keywords:** Eye abnormalities, Experimental models of disease

## Abstract

Ocular rosacea (OR) is a chronic inflammatory disease of the ocular surface frequently associated with meibomian gland dysfunction (MGD), with limited therapeutic options and an underexplored pathophysiology. Here, we uncover the pivotal role of mineralocorticoid receptor (MR) pathway overactivation in driving MGD and OR. Analysis of eyelid tissues from OR patients revealed increased MR expression and altered local corticosteroid metabolism, associated with inflammation, fibrosis, and impaired meibocyte renewal. Using a transgenic rat model overexpressing human MR, we demonstrate that MR overactivation initiates subclinical MGD and, with aging or ultraviolet-B exposure, drives a full OR-like phenotype characterized by gland dropout, oxidative and mitochondrial stress, immune infiltration, epithelial barrier disruption, and secondary corneal damage. Cross-species transcriptomic integration of rat, human MGD, and rosacea datasets identified a conserved MR-dependent gene signature, highlighting S100A9 as a specific downstream target and biomarker of MR activation. Local pharmacological MR antagonism suppressed S100A9 expression. These findings establish MR overactivation as a unifying pathogenic driver of MGD and OR and identify MR blockade as a promising therapeutic strategy, with S100A9 as a candidate biomarker for patient stratification and treatment monitoring.

## Abstract (web summary)

Ocular rosacea is a chronic inflammatory eye with limited treatment options. Here, the authors show that overactivation of the mineralocorticoid receptor pathway is a key driver of this condition, suggesting that blocking this pathway could be a promising treatment strategy.

## Introduction

Rosacea, a prevalent chronic inflammatory skin disease, affects up to 22% of the global population^1,2^. Approximately three-quarters of patients exhibit ocular surface involvement^3^ characterized by inflammation and neurovascular dysfunction of the ocular surface and eyelids associated with Meibomian gland dysfunction (MGD)^4^. The structural and functional alteration of Meibomian glands (MG) and the subsequent changes in the lipid composition of the tear film, result in corneal symptoms and alter the quality of life of patients with OR^5^. Eventually, limbal stem cell deficiency, neovascularization, loss of transparency and ulceration of the cornea can occur^3,6^. Despite its potentially blinding consequences, OR is often underdiagnosed and remains incurable, emphasizing the need for the identification of novel therapeutic targets^7^.

The pathogenesis of OR is multifactorial and remains incompletely understood. It involves aberrant innate and adaptive immune responses, neurovascular dysregulation^8–10^, neurogenic inflammation^8^ and fibrosis^11^. Activation of the toll-like receptor (TLR)^12^ and transient receptor potential (TRP) ion channels^13,14^ contribute to chronic inflammation of the ocular surface^15^. Environmental triggering factors, including ultraviolet (UV) exposure, psychosocial stress and glucocorticoids (GCs)^16,17^ contribute to OR and to MGD, which is a major component of the disease^18^. However, the precise mechanism by which GCs, despite their anti-inflammatory effects, act on OR and contribute to MGD is unknown.

Cortisol binds to the glucocorticoid receptor (GR) and to the mineralocorticoid receptor (MR) with similar high affinity. The metabolism of endogenous corticosteroids is controlled locally by hydroxysteroid dehydrogenase (11-βHSD) enzymes expressed in skin and eye tissues^19,20^. The low expression of 11-βHSD type 2 (11-βHSD2), which converts cortisol to inactive cortisone allows MR activation by GCs^21^, which could contribute to GC-induced OR. It has been demonstrated in previous studies that the oral administration of spironolactone, an MR antagonist, is an efficient treatment for skin rosacea^22^ and that it can reduce its incidence^23^. Furthermore, the topical administration of MR antagonists has been demonstrated to improve GC-induced epidermal atrophy^24^ and to enhance wound re-epithelialization in human skin treated with GC^25^. The administration of spironolactone drops increased the rate of corneal re-epithelialization in cases of wound healing delayed by GC^26,27^. Furthermore, mounting evidence suggests that MR overactivation induces inflammation, oxidative stress, fibrosis, vascular dysfunction and lipid dysmetabolism, all of which are observed in OR.

The subsequent investigation focused on elucidating the role of MR pathway overactivation in MGD and ocular surface damages relevant to OR.

## Results

### MR pathway is activated in eyelids from patients suffering from OR

**Fig. 1:**
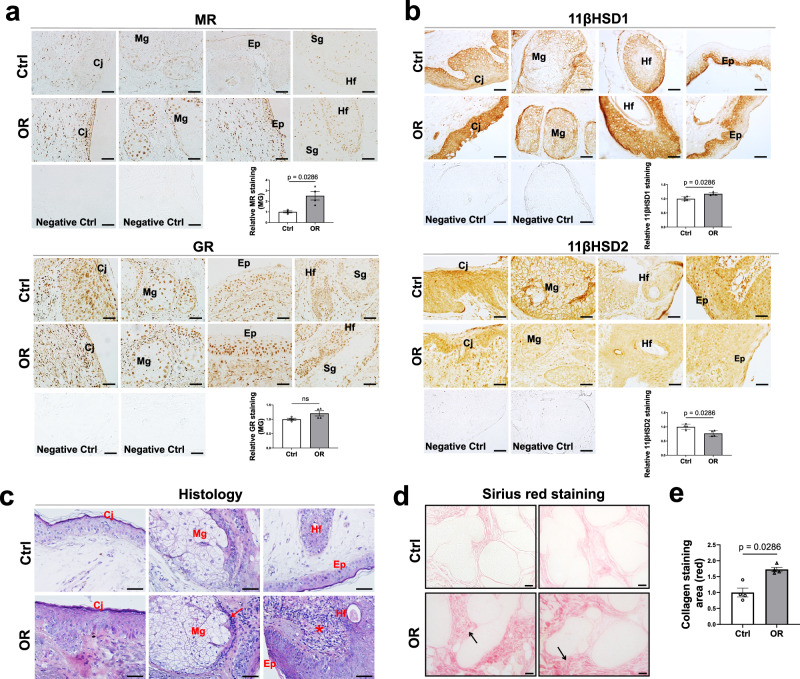
MR upregulation in OR patients is associated with histological and clinical pathologies of MGD. **a** Immunohistochemistry of MR and GR protein in eyelid tissues of patients with OR compared to control subjects. MR and GR protein levels in MGs determined by immunostaining and quantified by ImageJ (*n* = 4). **b** Immunohistochemistry of 11βHSD1 and 2 in eyelid tissues of patients with OR compared to control subjects. 11βHSD1 and 11βHSD2 protein levels in eyelid tissues determined by immunostaining was quantified by ImageJ (*n* = 4). **c** H&E staining of eyelid tissues from OR patients and control subjects (*n* = 4, 8 sections per patient). Infiltrating cells (arrow and asterisk). **d** Sirius red staining for collagen fibers (arrows) in MGs. **e** Collagen staining area in MGs quantified by ImageJ based on the same threshold of positive Sirius red staining (*n* = 4). Cj conjunctiva, Mg meibomian gland, Du ducts, Ep epidermis, Hf hair follicles, Sg, sebaceous gland. Scale bar: 100 µm. Data expressed as mean ± SEM. ns, *P* > 0.05 (Mann–Whitney *U* test with two-sided-comparisons). Source data are provided as a Source Data file.

There was no significant difference in age between patients with OR and controls (67.7 ± 11, 72 ± 19 years, *P* = 0.54) and there were 3 men and 1 woman in both groups. In the OR group, two patients presented rhinophyma, while the two others suffered only from OR. Immunohistochemistry of GR, MR, 11βHSD1 and 11βHSD2 was performed on the eyelids. In all the examined OR samples, MR immunostaining (see Fig. 1a) was enhanced in the nuclei of conjunctival epithelial cells, meibocytes, sebocytes, and epithelial cells as compared to controls while GR signal localization and intensity did not differ (Fig. 1a). 11βHSD1 was expressed in the cytoplasm of conjunctival epithelial cells, meibocytes, epithelial cells of hair follicles and basal cells of epidermis, while 11βHSD2 exhibited diffused staining in both the cytoplasm and nuclei of these cells. In the eyelid of OR samples, 11βHSD1 immunostaining was enhanced, while 11βHSD2 immunostaining was reduced (Fig. 1b), suggesting a potential local change in the cortisol/ cortisone ratio. The increase in the MR/GR and in the 11βHSD1/11βHSD2 ratio suggests that MR pathway is overactivated in the eyelids of patients with OR.

Histological analysis of eyelid from OR patients revealed hyperplasia of the conjunctiva and the epidermis and infiltration of inflammatory cells in the conjunctiva, around MGs and beneath the epidermis (Fig. 1c). The acini appeared smaller and hyper mature, suggesting abnormal meibocytes renewal (Fig. 1c). Sirius red that stains interstitial collagen indicated fibrosis within MG from patients suffering from OR (Fig. 1d, e).

### Overexpression of MR in young P1.hMR rats favours MGD initiation

**Fig. 2:**
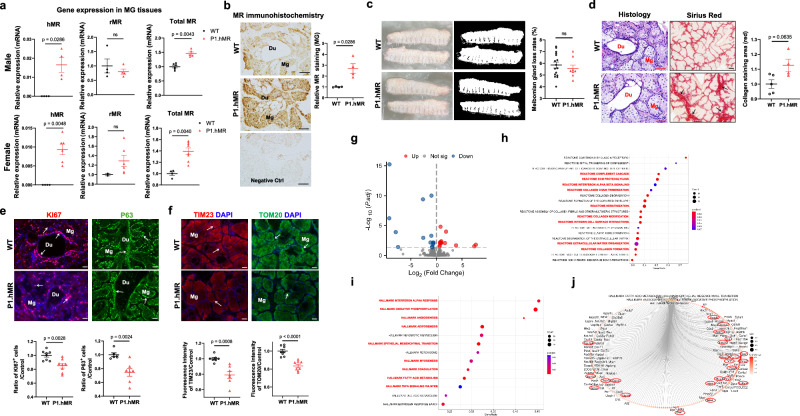
MR overexpression in the MG tissues of P1.hMR rats impairs meibocytes function. **a** hMR, rMR and total MR expression in MG tissues of P1.hMR and WT rats (*n* = 2–8 rats per experiment, the number in each experiment is represented by the individual dots in each graph) **b** MR immunostaining in the eyelid tissues of P1.hMR rats and WT was quantified by ImageJ (*n* = 4 rats, 4 sections per rat). **c** Macroscopic images of the inner eyelid and MGs from WT and P1.hMR rats. Meibomian gland loss area was quantified by ImageJ (WT: *n* = 16 rats, P1.hMR: *n* = 8 rats). **d** H&E and Sirius-Red staining (collagen fibers, arrows) in the MG of WT and P1.hMR rats. Collagen staining area quantified by ImageJ based on the same threshold of positive Sirius red staining (WT: *n* = 5 rats, P1.hMR: *n* = 4 rats). **e** KI67 (red colour, left) and P63 (green colour, right) stained cells in the MG of WT and P1.hMR rats (arrows). Ratio of positive immunosignal cells/total DAPI-marked cells was quantified by QuPath (*n* = 8 rats each group in KI67; WT: *n* = 6 rats, P1.hMR: *n* = 7 rats in P63). **f** TIM23 (red colour, left) and TOM20 (green colour, right) expression was quantified by ImageJ according to fluorescence intensity (*n* = 7 rats each group in TIM23; *n* = 8 rats each group in TOM20). Arrows indicate positively stained cells. **g** Volcano plot of genes differentially regulated by hMR overexpression in MG tissues of P1.hMR rats compared to WT rats (WT: *n* = 3 rats, P1.hMR: *n* = 2 rats). **h** GSEA using the RACTOME database reveals 20 significantly regulated pathways. **i** GSEA using the HALLMARK gene sets identifies 11 significantly regulated pathways. **j** Regulated HALLMARK pathways with highlighted genes associated with MGD and rosacea. Mg meibomian gland, Du ducts. Scale bar: 100 µm. Data expressed as mean ± SEM. ns, *P* > 0.05 (Unpaired *t* test with two-sided comparisons for (**c**, **e**, **f**). Multiple Mann–Whitney *U* test with two-sided comparisons for (**a**, **b**, **d**). Source data are provided as a Source Data file.

To investigate the contribution of MR overactivation to MGD and OR pathogenesis, we characterized the phenotype of the MG from P1.hMR rats of 6 months of age^28,29^. It was first confirmed that the human *NR3C2* (MR) transgene was expressed in the MGs (Fig. 2a) similarly in male and female P1.hMR rats. The total MR gene expression (hMR and rMR) exhibited a substantial increase in P1.hMR rats without altering endogenous rat MR and GR expression (Figs. 2a and S1a). P1.hMR rats exhibited enhanced MR staining in both the nucleus and cytoplasm in cells of MG (Fig. 2b) compared to WT rats, whereas no difference in GR immunostaining was observed in MG tissues between both groups (Fig. S1b), confirming MR overexpression and increased MR/GR ratio in P1.hMR rats, similar to the human OR tissues. In the MG of P1.hMR rats, MR immunostaining demonstrated a 2.6-fold increase in MR protein expression compared to WT rats (Fig. 2b).

Although no significant MG dropout and no histological alterations were observed in the in 6-month-old transgenic rats or (Fig. 2c, d) (Fig. S1c), incipient fibrosis (Fig. 2d) and signs of meibocytes renewal alterations were observed. The expression of KI67 and P63 in the basal layer of acini and of the ductal epithelium was indeed diminished in the MG of P1.hMR rats as compared to WT (Fig. 2e). In addition, a reduced expression of two mitochondrial proteins, TOM20 and TIM23 (Fig. 2f), could suggest an alteration of mitochondrial metabolism, which is supported by deregulation of the oxidative phosphorylation pathway in the transcriptomic analysis of MG from transgenic rats. At this age, these signs of meibomian gland dysfunction remained subclinical with no impact on lipid production or the ocular surface (Fig. S3).

The transcriptome of the MG from P1.hMR rats of 6 months of age identified 30 differentially expressed genes (DEG) with 14 upregulated and 16 downregulated genes as compared to WT littermates (Fig. 2g). Gene set enrichment analysis (GSEA) using REACTOME pathway database revealed pathways associated with the complement cascade, interferon signalling, keratinization, collagen and extracellular matrix organization (Fig. 2h). HALLMARK gene set highlighted oxidative phosphorylation, angiogenesis, epithelial mesenchymal transition, adipogenesis and fatty acid metabolism pathways (Fig. 2i), suggesting MGD initiation and alterations in biological functions relevant to the disease although no signs of OR was observed in 6-month old rats. Interestingly, these pathways share a common signature with a human MGD published transcriptome^28^. Genes of particular importance in the pathological pathways of MGD studies^29,30^ include *Vim, Pmel, Col3a1, Col1a1, Sdc4 and Fnln5*, which are associated with cell structure and the extracellular matrix; *Cyp1b1, Cyp1a1, Pdha1, Dld, Crat* and *Acsm3*, which are linked to metabolism and energy production; and *Kcnj8, Gas1, Wipf1* and *Stc1*, which are related to signal transduction (Figs. 2j and  S4). These genes were validated by quantitative PCR (qPCR) using MG tissues from an independent batch of P1.hMR and WT rats (Fig. S4). Additional genes of interest, including *Tlr3, Nos3, Cd36, Clu*, and *Ccl2*, identified in a published transcriptomic study on the skin of rosacea patients^31,32^, were also confirmed by qPCR in rat MG tissues (Fig. S4). These genes and related pathways are not specific to MGD and whether such deregulated genes play a role in MGD has not been studied but interestingly they were also deregulated in human tissues with MGD and skin rosacea. These results indicate that MR pathway overactivation in young rats initiate MGD causes transcriptional changes relevant to the molecular changes observed in human with MGD and skin rosacea.

### MR overexpression in old P1.hMR rats promotes MGD phenotype

**Fig. 3:**
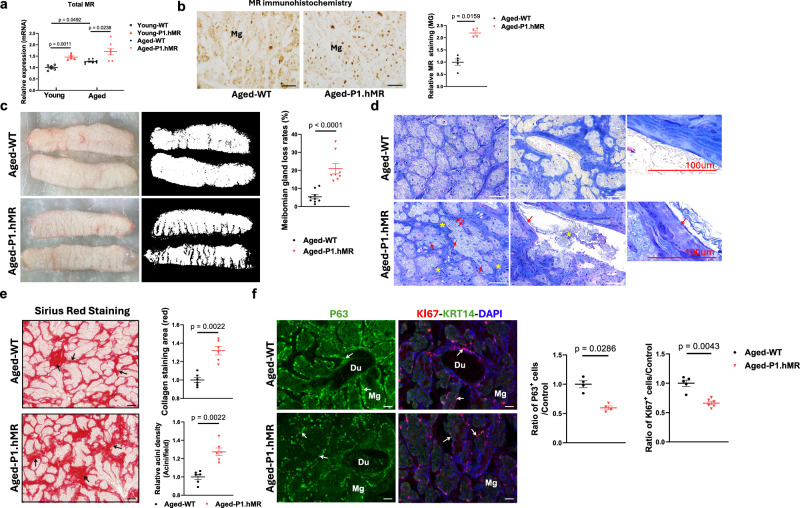
MR activation promotes MGD features in Aged-P1.hMR rats. **a** Total MR mRNA expression in MG tissues from young and aged WT and P1.hMR rats (*n* = 5–8 rats per experiment, the number in each experiment is represented by the individual dots in each graph). **b** MR (brown color) protein levels in the MG tissues of Aged-P1.hMR rats compared to Aged-WT were determined by immunostaining and quantified by ImageJ (Aged-WT: *n* = 5 rats, Aged-P1.hMR: *n* = 4 rats). **c** Macroscopic images of the inner eyelid and MGs from Aged-WT and Aged-P1.hMR rats. Meibomian gland loss area was quantified by ImageJ (Aged-WT: *n* = 9 rats, Aged-P1.hMR: *n* = 8 rats). **d** Representative semi-thin (1 µm) toluidine blue–stained sections of MG tissue from Aged-WT and Aged-P1.hMR rats (Aged-WT: *n* = 5 rats, Aged-P1.hMR: *n* = 4 rats). Arrows indicate hypermature nuclei and MG duct with features suggestive of keratinization, asterisks indicate increased collagen deposition between acini, along with possible fibrosis and structural disruption of the MG duct. **e** Sirius-Red staining for collagen fibers (arrows) in the MG of Aged-WT and Aged-P1.hMR rats. Collagen staining area in MGs quantified by ImageJ based on the same threshold of positive Sirius red staining. The density of acinar units was quantified by angiogenesis analysis in ImageJ and the number of complete meshes were calculated and compared (*n* = 6 rats). **f** P63 (green color, left) and KI67 (red color, right) expression in the MG of Aged-WT and Aged-P1.hMR rats. Arrows indicate positively stained cells. Protein expression level was determined by the ratio of positive immunosignal cells/ total DAPI-marked cells and quantified by QuPath (*n* = 4 rats each group in P63; Aged-WT: *n* = 5 rats, Aged-P1.hMR: *n* = 6 rats in KI67). Mg, meibomian gland; Du, ducts. Scale bar: 100 µm. Data expressed as mean ± SEM. ns, *P* > 0.05 (Kruskal–Wallis test with Dunn’s multiple comparisons in (**a**) and multiple Mann–Whitney *U* test with two-sided comparisons in (**b**, **c**, **e**, **f**). Source data are provided as a Source Data file.

MR signalling has been associated with aging in multiple tissues^33,34^. In the rat MG, total MR expression increased with age (6-months vs 12-months) but remained significantly higher in transgenic P1.hMR rats. (Fig. 3a). MR immunostaining confirmed enhanced MR protein in the nuclei of meibocytes in old P1.hMR rats (Fig. 3b). Quantification of the MG photographed  in ex vivo rat eyelids, similar to clinical in vivo meibography, revealed a loss of glandular surface area, demonstrating a clinical phenotype of MGD (Fig. 3c). Semi-thin sections of the MG showed fibrotic tissues surrounding the acini where hyper mature meibocytes and disorganized acini could be observed. Hyperkeratinization of the ducts caused their obstruction (Fig. 3d). Collagen staining further confirmed substantial fibrosis induced by MR overactivation (Fig. 3e). Functional degeneration identified by P63 and KI67 indicated critical role for MR in the meibocyte renewal and proliferation (Fig. 3f). The enhanced MGD phenotype in aged P1.hMR rats demonstrated that MR signaling plays a crucial role in age-related MGD pathology.

### MR overexpression exacerbates UVB-induced MGD in young P1.hMR rats mimicking OR

**Fig. 4:**
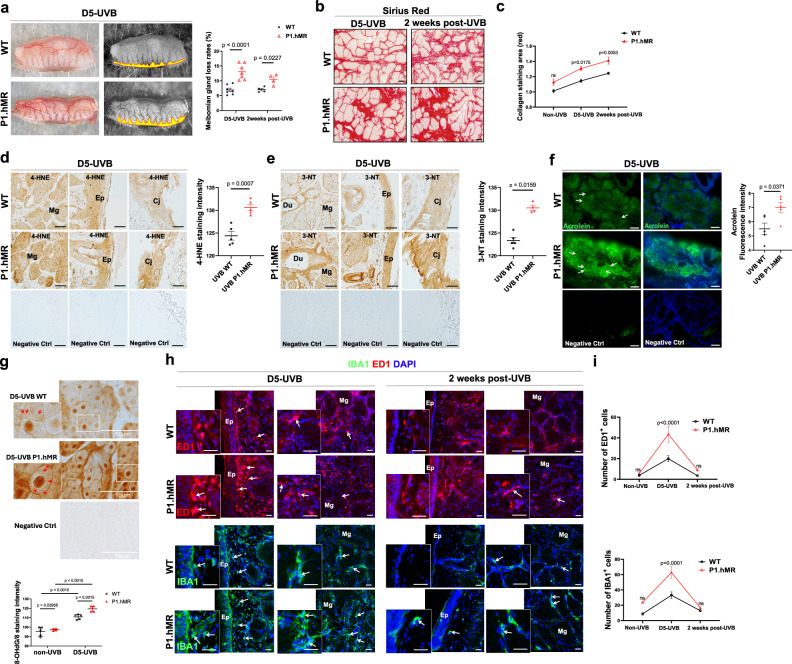
UVB-induced rat model mimicking OR is exacerbated by MR overexpression. **a** Representative macroscopic images of the inner eyelid and MGs after 5 days of UVB eyelid irradiation. MG loss area quantified by ImageJ (*n* = 7 rats each group in D5-UVB; *n* = 4 rats each group in 2 weeks post-UVB). **b** Sirius red staining on day 5 of UVB and 2 weeks post-UVB exposure. **c** Quantification of collagen area using ImageJ (WT: *n* = 5 rats, P1.hMR: *n* = 4 rats in non-UVB; WT: *n* = 4 rats, P1.hMR: *n* = 5 rats in D5-UVB; WT: *n* = 6 rats, P1.hMR: *n* = 5 rats in 2 weeks post-UVB). **d** 4-HNE immunostaining in eyelid tissues on day 5 of UVB quantified by ImageJ based on intensity (*n* = 5 rats). **e** 3-NT immunohistostaining on day 5 of UVB quantified by ImageJ based on intensity (*n* = 5 rats in UVB WT; *n* = 4 rats in UVB P1.hMR). **f** Acrolein immunostaining (green color) in MG tissues on day 5 of UVB quantified by ImageJ based on intensity (*n* = 5 rats). Arrows indicate positive acrolein staining, reflecting the presence of protein adducts formed during lipid peroxidation. **g** 8-OHdG/8 immunohistostaining in MG tissues on day 5 of UVB quantified by ImageJ based on intensity (WT: *n* = 5 rats, P1.hMR: *n* = 4 rats in non-UVB; *n* = 5 rats each group in D5-UVB). Arrows indicate areas of positive 8-OHdG immunohistochemical staining, indicative of oxidative DNA damage. **h** Infiltration of ED-1+ (red) and IBA1+ (green) cells in the eyelid tissues on day 5 and 2 weeks post-UVB. Arrows indicate infiltrating immune cells. **i** Quantification of positive-stained cells per field (WT: *n* = 6 rats, P1.hMR: *n* = 4 rats in non-UVB for ED1 and IBA1; WT: *n* = 8 rats, P1.hMR: *n* = 4 rats in D5-UVB for ED1 and IBA1; WT: *n* = 4 rats, P1.hMR: *n* = 5 rats in 2 weeks post-UVB for ED1; WT: *n* = 7 rats, P1.hMR: *n* = 5 rats in 2 weeks post-UVB for IBA1). Cj conjunctiva, Mg meibomian gland, Ep epidermis, Du duct. Scale bar: 100 µm. Data expressed as mean ± SEM. ns, *P* > 0.05 (two-way ANOVA with Fisher’s LSD post hoc test in (**a**, **g**). Two-way ANOVA with Tukey’s multiple comparisons in (**c**, **i**); Mann–Whitney *U* test with two-sided comparisons in (**d**–**f**). Source data are provided as a Source Data file.

As UVB exposure is a triggering factor for OR, and ocular surface defects associated with MGD are key features of the disease, a rat model of UVB-induced MGD and OR was developed using a selective and short exposure of rat eyelids to high-dose UVB for five consecutive days^35^. It was applied in 3-month-old rats to evaluate whether MR overexpression aggravates the UV response. Following UVB exposure, the MG swelling and dropout and palpebral conjunctival hyperemia were exacerbated in P1.hMR rats as compared to WT, with a greater MG loss area quantified (Fig. 4a). Progressive fibrosis was observed in the MG from day 5 to 2 weeks after UVB exposure, with more extensive fibrosis in P1.hMR rats compared to WT rats (Fig. 4b, c). Oxidative stress markers were exacerbated in P1.hMR rats as shown by a more pronounced staining of 4-hydroxynonenal (4-HNE), a marker for lipid peroxidation, and 3-nitrotyrosine (3-NT), a marker for nitrosative stress in the MG, epidermis and conjunctiva (Fig. 4d, e). Enhanced expression of acrolein, a non-specific marker of mitochondria-driven oxidative injury, further supported enhanced lipid peroxidation in MG of P1.hMR rats (Fig. 4f). In addition, 8-OHdG, a marker for oxidative DNA damage was significantly more pronounced in both the nuclei and mitochondria of meibocytes in P1.hMR rats compared with WT, suggesting that MR overexpression enhances the susceptibility of meibocytes to oxidative stress, particularly mitochondrial DNA damage and dysfunction (Fig. 4g). The transient inflammatory response observed on day 5 of UVB exposure was more severe in the transgenic rats, with increased infiltration of dendritic cell and monocytes/macrophages (ED1 and IBA1 positive) beneath the epidermis, within the connective tissue between MG acini and along the ducts in P1.hMR rats (Fig. 4h, i).

**Fig. 5:**
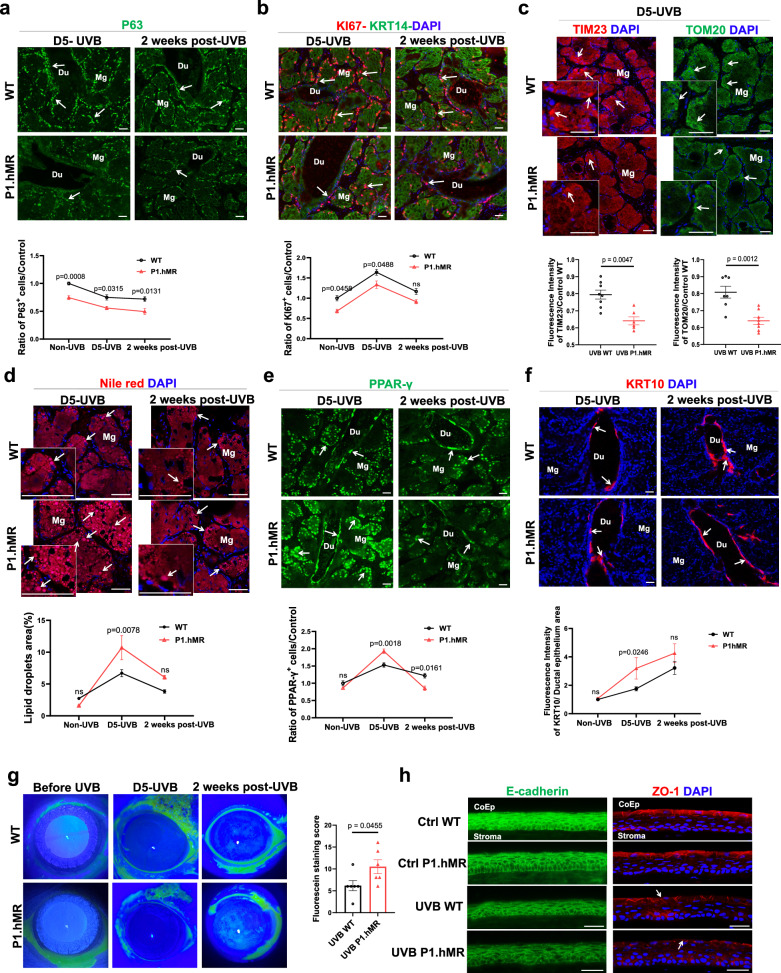
MR overexpression aggravates the UVB-induced MGD. **a**–**f** Analysis of MG on day 5 of UVB and 2 weeks post-UVB, **a** P63 immunostaining quantified by QuPath (ratio of positive cells/ total DAPI-marked cells) (WT: *n* = 6 rats, P1.hMR: *n* = 5 rats in non-UVB; WT: *n* = 5 rats, P1.hMR: *n* = 4 rats in D5-UVB; *n* = 4 rats each group in 2 weeks post-UVB). **b** KI67 (red) and KRT14 (green) immunostaining quantified by QuPath (ratio of positive cells/total DAPI-marked cells) (WT: *n* = 5 rats, P1.hMR: *n* = 4 rats in non-UVB and 2 weeks post-UVB; WT: *n* = 6 rats, P1.hMR: *n* = 4 rats in D5-UVB). **c** TIM23 (red) and TOM20 (green) immunostaining quantified by ImageJ (*n* = 8 rats in UVB WT, *n* = 6 rats in UVB P1.hMR for TIM23; *n* = 7 rats in UVB WT, *n* = 8 rats in UVB P1.hMR for TOM20). **d** Nile red staining (red) quantified by ImageJ (lipid droplet area with a consistent threshold) (WT: *n* = 7 rats, P1.hMR: *n* = 6 rats in non-UVB; *n* = 4 rats each group in D5-UVB; WT: *n* = 5 rats, P1.hMR: *n* = 4 rats in 2 weeks post-UVB). **e** PPAR-γ (green) immunostaining quantified by QuPath based (ratio of positive cells/total DAPI-marked cells) (WT: *n* = 5 rats, P1.hMR: *n* = 4 rats in non-UVB; WT: *n* = 8 rats, P1.hMR: *n* = 5 rats in D5-UVB; WT: *n* = 6 rats, P1.hMR: *n* = 4 rats in 2 weeks post-UVB). **f** KRT10 (red) immunostaining quantified by ImageJ (WT: *n* = 5 rats, P1.hMR: *n* = 8 rats in non-UVB; WT: *n* = 7 rats, P1.hMR: *n* = 8 rats in non-UVB; WT: *n* = 6 rats, P1.hMR: *n* = 4 rats in 2 weeks post-UVB). **g** Corneal epithelial defects evaluated by fluorescein staining quantification (*n* = 6 rats). **h** E-cadherin and ZO-1 in corneal epithelium at 2 weeks after UVB irradiation on eyelids compared to non-UVB control group (*n* = 4–6 rats). Mg meibomian gland, Du ducts, CoEp corneal epithelium. Arrows indicate stained cells or area. Scale bar: 100 µm. Data expressed as mean ± SEM. ns, *P* > 0.05 (two-way ANOVA with Tukey’s multiple comparisons in (**a**, **b**, **d**, **e**). Two-way ANOVA with Fisher’s LSD post hoc test in (**f**); Mann–Whitney *U* test with two-sided comparisons in (**c**, **g**). Source data are provided as a Source Data file.

MR overexpression in P1.hMR rats further impaired MG stem cell function and proliferation, accompanied by mitochondrial dysfunction, compared to WT rats in the UVB-induced MGD and OR model. On day 5 of UVB exposure, markers of MG stem cell marker, P63, showed a continuous decrease up to two weeks (Fig. 5a), and the transient rise in KI67-positive proliferating meibocytes observed on day 5 was further attenuated at two weeks post-UVB in P1.hMR rats (Fig. 5b), indicating impaired meibocytes renewal. Furthermore, TOM20 and TIM23 were reduced in P1.hMR rats on day 5 of UVB exposure compared to WT rats (Fig. 5c).

All the pathological changes induced by UVB contribute to the development of MGD, which is further aggravated by MR overexpression. On day 5 of UVB exposure, markers of lipid synthesis dysregulation were significantly exacerbated in P1.hMR rats, with increased accumulation of intracellular lipid droplets in meibocytes (Fig. 5d) and elevated PPARγ expression in both meibocytes and ductal epithelial cells. The later then declined more severely by two weeks post-UVB in P1.hMR rats compared to WT (Fig. 5e). While the hyper-keratinization was induced by the UVB irradiation on eyelids, a more significant increase was observed in P1.hMR rats compared to WT (Fig. 5f), indicating more severe functional damage exacerbated by hMR overexpression in P1.hMR rats.

### Corneal damage secondary to MGD was induced by MR overexpression

No spontaneous clinical alterations of the cornea were detected in P1.hMR rats up to 6 months of age (Fig. S5). At baseline and after 5 days of eyelid-targeted UVB exposure, no fluorescein staining was observed in either P1.hMR or WT rats, as the cornea was carefully shielded from direct irradiation during this period. However, by two weeks following UVB exposure, diffuse punctate fluorescein staining—indicative of punctuate keratitis, likely secondary to UVB-induced MGD—was observed, with more widespread staining in P1.hMR rats compared to WT (Fig. 5g). Corneal epithelial integrity was also more severely compromised in P1.hMR rats, as evidenced by greater disorganization of the cell junction proteins, E-cadherin and ZO-1 (Fig. 5h).

### Gene dysregulation in the MG in response to UVB radiation is highly relevant to OR

**Fig. 6:**
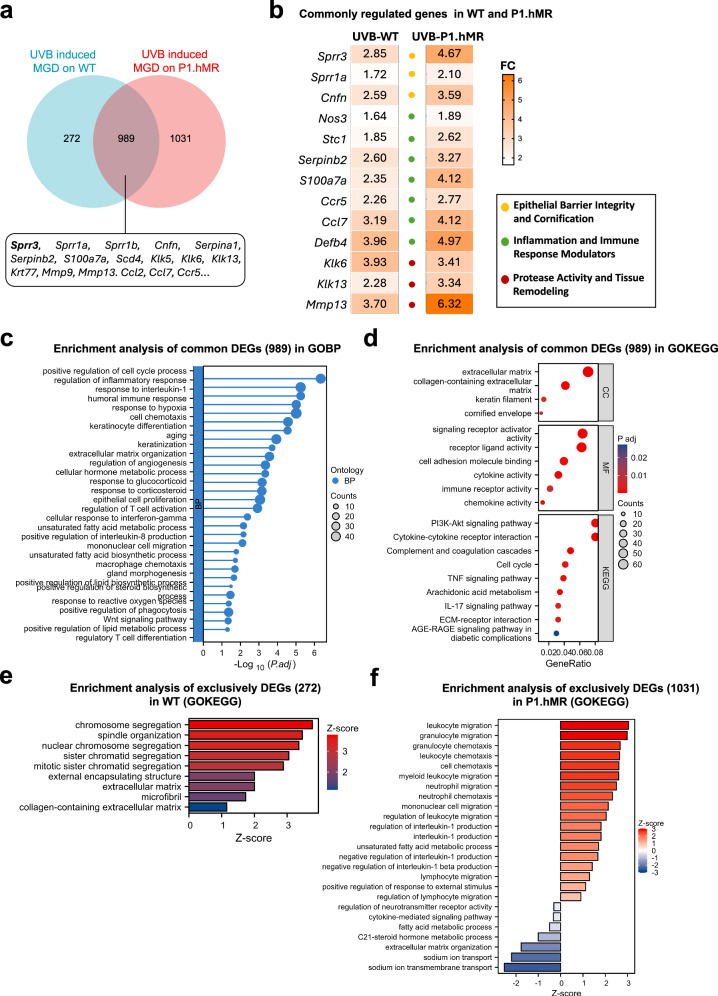
Transcriptomic analyses of UVB-induced MGD in WT and P1.hMR rats. **a** Venn diagrams illustrating differentially expressed genes (DEGs) in the MG of WT and P1.hMR rats exposed to UVB. DEGs critically associated with MGD and rosacea are listed. **b** Fold Change (FC) values of representative common DEGs in UVB-WT (UVB WT vs. non-UVB WT) and UVB-P1.hMR (UVB P1.hMR vs. non-UVB P1.hMR) rats. **c** Enrichment analysis of common DEGs using GOBP database. **d** Enrichment analysis of common DEGs using KEGG database. **e** Enrichment analysis of DEGs exclusively identified in WT rats. **f** Enrichment analysis of DEGs exclusively identified in P1.hMR rats. *n* = 3–5 rats in each group.

After 5 days of UVB exposure, 1271 DEGs were found in the MG of WT rats (GSE291177), including 611 upregulated and 660 downregulated genes. In contrast, 2040 DEGs were detected in the MG of P1.hMR rats (GSE291177), with 920 upregulated and 1120 downregulated genes. Intersection analysis revealed 989 genes shared between P1.hMR and WT, while 1031 genes were exclusively regulated in P1.hMR rats and 272 genes were specific to WT rats (Fig. 6a).

Among the shared DEGs, those associated with epithelial integrity, immune responses, collagen production and tissue remodelling showed greater fold changes in P1.hMR rats (Fig. 6b). Gene ontology enrichment analysis of these common DEGs indicated involvement in biological processes such as cell cycle, inflammatory and humoral immune response, keratinization, extracellular matrix organization, angiogenesis, hormone metabolism, response to corticosteroid, unsaturated fatty acid metabolism, gland morphogenesis, Wnt signalling, and lipid metabolism (Fig. 6c). Cellular component analysis highlighted enrichment in collagen and extracellular matrix regulation, keratin filaments, and cornified envelope pathways. KEGG pathway analysis further identified significant regulation of PI3K-Akt signalling, complement and coagulation cascades, TNF signalling, arachidonic acid metabolism, IL-17 signalling, and ECM-receptor interaction (Fig. 6d). Thus, using UVB irradiation, the main triggering factor of OR, to induce MGD effectively reflects the pathogenic mechanism of OR.

### MR overexpression amplifies inflammatory responses and dysregulation of proliferation and differentiation in the UVB-induced OR model

Enrichment analysis of UVB-induced DEGs exclusively identified in the MG of WT rats showed pathways related to cell cycle and proliferation, and collagen-extracellular matrix organisation (Fig. 6e), indicating activation of repair mechanisms. In contrast, genes exclusively regulated in P1.hMR rats associated with inflammatory responses, unsaturated fatty acid and steroid hormone metabolism, extracellular matrix organization, neurotransmitter receptor and sodium ion transport (Fig. 6f). These distinct transcriptomic changes in P1.hMR rats further support the role of MR overactivation in amplifying inflammation, neuroimmune responses, metabolic dysregulation and ion transport imbalance, all of which likely contribute to the exacerbation of OR.

**Fig. 7:**
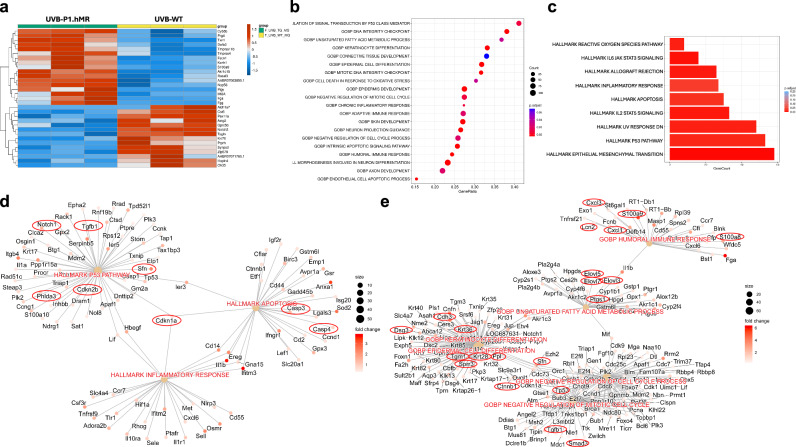
Transcriptomic analysis of hMR overexpression in UVB-induced MGD and OR model. **a** Heatmap shows 17 significant upregulated genes and 14 downregulated genes in UVB-P1.hMR vs UVB-WT MGs. **b** Enrichment analysis shows significantly regulated pathways using GOBP gene set. **c** Enrichment analysis shows significantly regulated pathways using HALLMARK gene set. **d** Gene concept network shows significantly regulated gene sets using HALLmark database. **e** Gene concept network shows significantly regulated gene sets using GOBP database.

In addition, MR overexpression induced a different transcriptomic signature in the UVB-induced MGD and OR model (UVB-P1.hMR vs. UVB-WT), compared to that observed under control conditions (P1.hMR vs. WT), suggesting that the regulatory role of MR is modulated by UVB-induced stress (Fig. 7a). Enrichment analysis of DEGs induced by MR overexpression under UVB model showed pathways related to cell proliferation, keratinocytes and epidermal differentiation, and apoptosis using GOBP and HALLMARK gene sets (Fig. 6b, c). Genes that inhibit proliferation and promote apoptosis include *Tgf-β1*, *Notch1*, *Phlda3*, *Cdkn1a*, *Cdkn2b*, *Casp3*, *Casp4*, and *Tp53*. Genes that facilitate keratinocyte and epidermal differentiation include *Sprr3*, *Krt28*, *Krt36*, *Tgm1*, *Ppl*, *Dsg3*, and *Cdh3* (Fig. 6d, e). *S100a8*, *S100a9*, *Lcn2*, *Cxcl1*, and *Cxcl3*—genes significantly associated with MGD and rosacea—were upregulated as part of the humoral immune response. Genes involved in fatty acid metabolism, including *Elovl3*, *Elovl5*, *Elovl7*, and *Ptgs1*, were also upregulated by MR overexpression. (Fig. 6e). The transcriptomic results supported the MGD-induced OR phenotypes in young P1.hMR exposed to UV.

### S100a9 is a specific MR target, associated with MGD and skin rosacea

The transcriptome of MGs from P1.hMR rats on day 5 of UVB exposure was compared to transcriptomic profiles of human MGs with MGD^28^ (GSE17822) and of human skin with rosacea^36^ (GSE65914). A notable overlap in gene regulation was observed between the P1.hMR rat model and MGD patients (Table S1), as well as between P1.hMR rats and human rosacea skin (Table S2), providing further support for the involvement of MR pathway activation in the pathogenesis of MGD and rosacea in humans (Fig. S6).

**Fig. 8:**
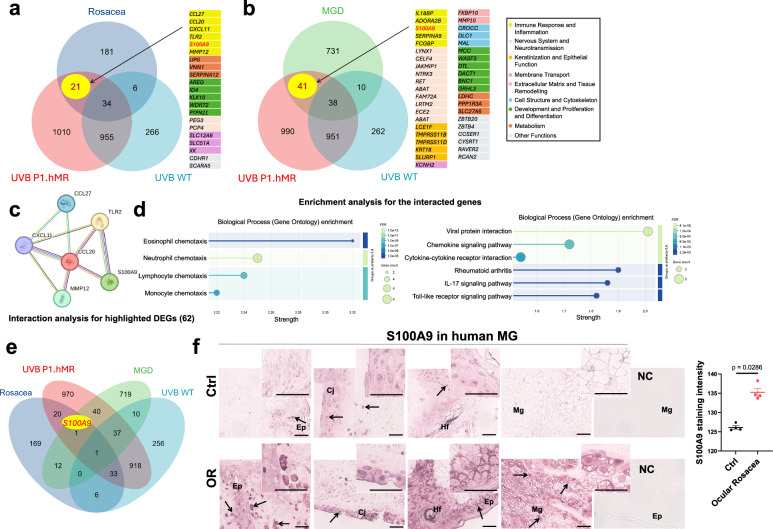
Multi-omics analyses identify S100A9/S100a9 as a key mediator of MR-exacerbated pathogenesis of MGD in Ocular Rosacea. **a** Venn diagram showing the intersection of DEGs in the skin of rosacea patients (GSE65914), UVB-induced MGD in P1.hMR rats (UVB P1.hMR), and UVB-induced MGD in WT rats (UVB WT). Twenty-one genes specific to MR overactivation are commonly regulated in human skin rosacea. **b** Venn diagram showing the overlap of DEGs in the MG from MGD patients (GSE17822), UVB-induced MGD in P1.hMR rats (UVB P1.hMR), and UVB-induced MGD in WT rats (UVB WT). Forty-one genes specific to MR overactivation are commonly regulated in human MGD. **c** Network analysis reveals interaction among DEGs associated with rosacea, MGD, and MR overexpression. The highlighted strings indicate existing and potential interactions between genes. **d** Gene Ontology Biological Process (GOBP) and KEGG pathway analysis of *S100A9* and its interacting genes. **e** Venn diagram with the 4 databases identifies *S100A9*/*S100a9* as a gene associated with MGD and rosacea and specifically linked to MR overexpression. **f** Immunohistochemistry and quantification of S100A9 protein expression in human MGs tissues from OR patients and control subjects (*n* = 4). Arrows indicate positive staining. Cj conjunctiva, Mg meibomian gland, Ep epidermis, Hf hair follicles. Scale bar: 100 µm. Data expressed as mean ± SEM. (Mann–Whitney *U* test with two-sided-comparisons). Source data are provided as a Source Data file.

Additional cross-analyses were performed between the DEGs specific to P1.hMR rats and the two human transcriptomic datasets^28,36^. A total of 21 genes specifically regulated by MR overaction in the P1.hMR rat model overlapped with genes deregulated in human rosacea skin (Fig. 8a), while 41 MR-regulated genes in P1.hMR rats were shared with those found in human MGD (Fig. 8b). Among these genes, *CCL27, CCL20, CXCL11, TLR2, S100A9* and *MMP12* were identified as an interaction network focus on immune cells chemotaxis, IL-17 and Toll-like receptor signaling, involved in rosacea and MGD (Fig. 8c, d)^37–39^. Further comparison between these two gene sets identified a single gene, *S100A9* (Fig. 8e), as a common marker of MR activation in both skin rosacea and MGD. S100A9 immunohistochemistry was further evaluated in human eyelid tissues. In control subjects, S100A9 was primarily located in the epithelium and in cells that may correspond to immune cells beneath the epidermis. In patients with OR, S100A9 expression was not only markedly increased in the epithelium and immune cells, but was also highly expressed in meibocytes in the MG (Fig. 8f). These findings provide compelling evidence supporting the role of MR dysregulation in the pathophysiology of oculo-cutaneous rosacea and highlight S100A9 as a potential biomarker of MR pathway activation in both MGD and OR.

### Spironolactone drops favoured ocular surface integrity and downregulated *S100a9*

Corneal surface damage, including epithelial barrier disruption^40^, limbal stem cell deficiency and neovascularization, delayed wound healing, is a major source of morbidity in moderate to severe cases of OR, and can also occur as a consequence of MGD^3,6^. Previously we have proved that spironolactone (SPL), a potent MR antagonist (MRA) could prevent corneal neovascularization (CoNV) and improve corneal re-epithelialization in corneal limbal stem cell deficiency (LSCD) and wound healing model^27,41^. Transcriptomic and enrichment analysis (GSE245468) revealed that MR antagonism suppressed inflammation and EMT and, upregulated epithelial cell proliferation (Fig. S7a–c and Supplementary Data 1). Spironolactone efficiently downregulated *S100a9* expression in the ocular surface tissues (Fig. S7d). These findings confirmed that *S100a9 is a specific* MR target gene.

## Discussion

We provide herein a set of arguments supporting the hypothesis that excessive activation of the MR pathway contributes directly to both the initiation and exacerbation of MGD and OR. In addition, S100A9 has been identified as a biomarker of MR pathway activation in OR.

MR overexpression was observed at the site of pathology in the eyelid and ocular surface tissues from patients with OR, associated with inflammatory cell infiltration and fibrosis in the MGs, consistent with pathological features reported in severe cutaneous rosacea^42^. The exact mechanisms of MR activation remain to be determined. It could be related to the overexpression of the receptor, abnormal local metabolism of corticoids and/or to ligand-independent MR activation. Whether cortisol is increased locally in the MG and eyelids cannot be extrapolated solely by the 11βHSD activity and should be further explored by in situ steroidome analysis.

Signs of abnormal acinar renewal and differentiation likely contribute to the development of MGD in OR patients^43,44^. In the posterior segment of the eye, it was shown that local MR pathway activation in retinal and choroidal cells induced low-grade inflammation, oxidative stress, fibrosis and neovascularization^45^. In systemic pathogenic mechanisms such as hypertension, wound healing, inflammation or metabolic diseases, a pathogenic role of MR hyperactivation has been demonstrated in vascular endothelial and smooth muscle cells^46^. But local pathogenic activation in specific organs or tissues has also been demonstrated, such as in adipose tissue, where it has been shown to contribute to adipocyte dysfunction and subsequent metabolic disorders^47^ or in the heart, where MR-mediated damages were shown in cardiomyocytes using transgenic mouse models^48^.

The generation of a transgenic rat overexpressing hMR (P1.hMR rats) provided further insight in the role of MR in the pathogenesis of MGD and OR. In the 6-month-old transgenic rats, signs of MGD initiation including decline in meibocytes renewal and mitochondria dysfunctions were observed and the MG transcriptome identified pathways related to oxidative phosphorylation and peroxisome, which is consistent with the oxidative dysregulation observed in patients with MGD^49^. Since P1.hMR rats express hMR in all MR-expressing organs, it cannot be excluded that the initiation of MGD could result from MR overactivation both locally and systemically, although no signs of heart or kidney dysfunction was detected in these animals under basal conditions. The expression of hMR in innate immune cells could also contribute to the pathogenesis of the disease in the rat model and in humans. A transgenic animal model in which hMR expression was limited to the MG would be interesting, but it would not have more accurately mimicked the human disease, which is known to be multifactorial and to involve systemic immune and inflammatory factors.

Both aging and UV exposure induced clinical signs of MGD and related OR in P1.hMR rats. In several organs, MR activation has been shown to contribute to pathological aging. In the heart, MR expression was elevated with aging in rodents and associated with increased expression of cell cycle arrest markers (p16, p21, p53), mitochondrial dysfunction, and oxidative stress^33^. In the skin, epidermal MR expression increased with age and contributed to dermal atrophy and extracellular matrix (ECM) remodeling^50^. In adipocytes, specific MR overexpression caused mitochondrial dysfunction, oxidative stress, and premature cellular senescence^51^. Notably, MR blockade has been shown to attenuate these aging-associated features. Consistent with these findings, MR was overexpressed in the MG in 1-year-old P1.hMR rats. It was associated with MGD phenotype, characterized by significant MG dropout, alterations in meibocytes, and fibrotic remodeling of the MG. These changes were accompanied by further impaired renewal and proliferative capacity of meibocyte progenitor cells, suggesting that MR signaling contributes to MGD pathogenesis through regulation of fibrosis, meibocyte proliferation, and maturation. Indeed, previous studies have shown that reduced proliferation of meibocyte progenitor cells caused the hypermaturation of meibocytes and, ultimately, a decline in gland function in age-related MGD^52^. UV exposure, which is the primary triggering factor of OR, exacerbated the clinical, histological and transcriptomic manifestations of MGD and OR in P1.hMR. The pathological features resemble the ones described in human tissues with OR, in which signs of MR overactivation have been evidenced. A greater number of genes related to inflammation, oxidative stress, epithelial integrity and tissue remodelling were regulated in the transgenic rats compared to in WT. More specifically, the upregulation of *Sprr3* may drive aberrant keratinization, a key pathogenic mechanism in MGD^28^ and regulate IL-33 which amplifies inflammation^53^ and tissue remodelling^54^. In hMR-overexpressing rats, the higher *Nos3* expression may lead to excessive nitric oxide (NO), contributing to vasodilation and the development of eyelid telangiectasia^55^. Among the DEGs uniquely regulated in P1.hMR rats after UVB exposure, several are involved in chemotaxis, IL-1 signalling, immune cell infiltration, fatty acid metabolism, and ion channel activity—pathways known to promote inflammation and clinical features of rosacea^56^. Notably, *S100a9*, a direct MR target, was highly upregulated and is known to activate TLR4 and RAGE, amplifying inflammatory responses^57^. Additionally, the regulation of neurotransmitter receptor activity and sodium ion transport suggests MR involvement in neurovascular and neurogenic mechanisms, consistent with the role of neuropeptides and TRP channels in OR^58,59^. These findings support MR overactivation as a driver of immune, metabolic, and neurogenic dysregulation in OR.

Transcriptomic analysis of MR overexpression under UVB induction, compared to the WT-UVB group, revealed that MR is involved in regulating glandular epithelial cell proliferation and differentiation in response to UVB stress. These findings are consistent with those observed in age-related MGD with MR overactivation. Proliferation contributes to MG stem cell renewal, which is essential for maintaining MG function and meibum secretion, while aberrant differentiation leading to keratinization is a key pathological feature of MGD^43^. Indeed, MR has emerged as a modulator of functional maturation and survival in various stem and epithelial cell types, as demonstrated in other models where MR signaling was shown to alter neuronal maturation, cardiomyocyte contractility, and adipocyte differentiation^60^, contributing to tissues homeostasis or pathology depending on specific cell type, context and activation state. In the ocular surface tissues, MR overexpression restrained the MG stem cell renewal and promoted the hypermaturation and keratinization, while MR antagonist facilitated the corneal epithelial cell proliferation with suppression of inflammatory response following UVB damage. These results highlight the role of MR signaling in maintaining the balance of proliferation and differentiation in response to stress, and identifies MR as a player in epithelial and glandular biology of ocular surface tissues.

Intersection analysis revealed that nearly half of the genes commonly regulated in P1.hMR rats and in human MGD or cutaneous rosacea are associated with MR signalling, underscoring its central role in disease pathogenesis. Key genes such as *CCL27, CCL20, CXCL11, TLR2, S100A9*, and *MMP12* form an interaction network involved in chemotaxis, IL-17, and Toll-like receptor signalling—pathways known to drive rosacea-related inflammation^37,38^. Additionally, the MR-regulated gene *SLC27A6*, which mediates long-chain fatty acid uptake, may influence meibomian gland lipid composition, a hallmark of MGD^61^. These findings further confirmed the implication of MR overactivation in immune, metabolic, epithelial, and neurogenic mechanisms that contribute to both MGD and oculo-cutaneous rosacea.

The search for MR-specific downstream effectors identified S100A9 as a potential key mediator of MR-driven pathology in MGD and OR. Indeed, S100A9 promotes inflammation via TLR4/MyD88 signalling, induces M1 macrophage polarization through TLR4/NF-κB^38,62^, enhances neutrophil chemotaxis, and drives keratinization^28,63^, all processes implicated in rosacea pathogenesis. In OR patient tissues, S100A9 expression was markedly increased in immune cells, the conjunctival epithelium, and MR-overexpressing meibocytes. Given its role in amplifying oxidative stress and inflammation^64,65^, S100A9 could contribute to MG damage and dysfunction. But the exact role of S100A9 should be further evaluated using specific deletion of S100A9 in MGD animal models.

Notably, *S100a9/S100A9* was the only gene both MR-specific in the rat OR model and associated with human MGD and OR pathology. Its expression is also downregulated by spironolactone, an MR antagonist, in a LSCD model, relevant to corneal pathology in OR^41^, supporting its regulation by MR. Additionally, *S100a9* is upregulated in MR-overexpressing mice with choroidal neuropathy, reinforcing its role as MR targets in ocular tissues^66^. As S100A9 is detectable in tears and correlates with MGD severity in dry eye disease^67^, it may serve as a non-invasive biomarker of MR activation in OR, offering a potential tool for patient stratification and MR-targeted therapies.

This study has some limitations. The therapeutic effects of MR inhibition were studied in a corneal model relevant to OR to enable a more robust and consistent evaluation. In the UVB-induced OR model developed in P1.hMR, it was not feasible to test spironolactone drops as eyelid edema prevented the proper delivery of the drug and therefore whether local MR antagonism could alleviate MGD and OR will be evaluated in further clinical studies. In the cornea model, we could demonstrate the specificity of *S100a9* expression regulation by MR activation. But the exact pathogenic role of S100A9 still remain to be investigated. Nonetheless, rodent models cannot fully recapitulate the human clinical spectrum of OR. To directly assess MR involvement in human disease, we have developed topical spironolactone eye drops, previously validated for safety; however, clinical efficacy data are not yet available.

**Fig. 9:**
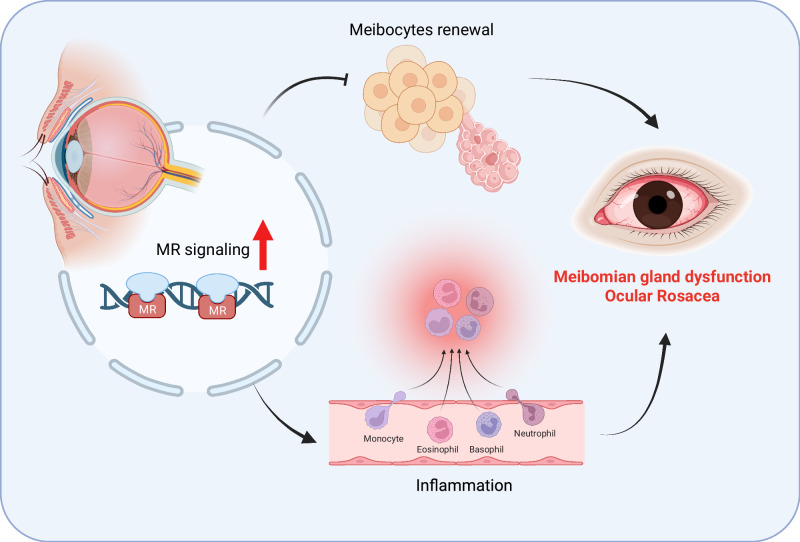
Involvement of MR in MGD and OR. MR overactivation impairs meibocyte renewal and promotes inflammation, driving MGD and OR. Created in BioRender. Zhu, Z. (2026) https://BioRender.com/q0j0614.

In conclusion, our findings demonstrate that MR pathway activation contributes to the pathogenesis of MGD and OR. Upregulation of MR signaling on the ocular surface exacerbates MGD and rosacea by promoting inflammatory responses and impairing meibocyte renewal and function (Fig. 9). Accordingly, MR antagonism represents a promising therapeutic strategy, while S100A9 may serve as a biomarker to identify patients most likely to benefit from targeted intervention. Future clinical trials are warranted to confirm the therapeutic utility of MR blockade in MGD and OR using either systemic or local treatments.

## Methods

### Human eyelid samples collection

Patients and control subjects were diagnosed by both an ophthalmologist and a dermatologist OR was diagnosed based on ocular symptoms and clinical signs of OR including lid margin telangiectasia, blepharitis, blepharoconjunctivitis, and meibomian gland dysfunction, based on examination of the MG orifices, irregularity of the palpebral margin, meibomian gland loss and abnormal vascularization of the eyelid margins. Inflammatory conditions other than OR were excluded. The surgical pieces obtained as surgical waste from blepharoplasty surgery, were immediately fixed in 10% formalin for 24 h, dehydrated, embedded in paraffin wax and cut into 7-µm thick slices and stained with hemalum-eosine, for histological analysis. Diagnosis of OR patients and control subjects were further confirmed by a pathologist on the sections. Additional sections were used for specific staining relative to the present study (see below). We analyzed the tissue sections from 4 patients with OR (3 men and 1 woman, 70 years, 71 years, 52 years, 78 years) and 4 control subjects without ocular or skin rosacea (3 men and 1 woman 44 years, 90 years, 78 years, 76 years). The collection and storage of human biological samples were approved by French ethics committee CCP Ile de France 1 (no. 2016-nov-14390). All patients signed written consent for non-opposition to the use of surgical waste in research in accordance with French regulations governing the use of human biological materials.

### Animal experiments

All experiments were performed in accordance with the European Communities Council Directive 86/609/EEC and French national regulations and approved by local ethics committee (# 23478-2020010317557546 v4, Charles Darwin). To study the role of MR in the pathology of OR, we generated P1.hMR rats on Sprague-Dawley genetic background. The human MR (hMR) gene is expressed under the control of the proximal P1 promoter of the *NR3C2*^48,68^. The P1 promoter drives the expression of hMR in all MR-expressing tissues, although the strength of its transcriptional activity varies among organs^69^. Using metabolic cages, we verified that up to one year of age, the P1.hMR rats did not develop kidney failure and no sign of MR-induced cardiac dysfunction was detected at the molecular level in the heart. Overexpression of *NR3C2* expression was validated in ocular tissues of rats in both male (WT: *n* = 3–6 rats; P1.hMR: *n* = 3–8 rats) and female (WT: *n* = 2–4 rats; P1.hMR: *n* = 6–8 rats) by transcriptomic and qPCR analysis. Other animals (WT: *n* = 4–6 rats; P1.hMR: *n* = 4–6 rats) were used for characterization in morphology and pathology. All animals were kept in pathogen-free conditions with free access to food and water and housed in temperature-controlled room with a 12-h light/12-h dark cycle. Anaesthesia was induced by intraperitoneal ketamine 100 mg/kg and Xylazine 10 mg/kg. Animal were euthanized by intraperitoneal injection of fatal dose of Euthasol® Vet.

### UVB irradiation on rat eyelids

The upper eyelids of rats were shaved and exposed to 1000 µW/cm^2^ of UVB (312 nm, Herolab, Wiesioch, Germany) for 5 min (300 mJ/cm^2^) daily for 5 days^35^. The cornea and the whole body were covered and protected. Lesions of eyelids were photographed under the microscopy from day 0 to day 5. Rats were euthanized after 5 days to evaluate the acute damages of ocular surface tissues and MR-related pathology (WT: *n* = 5 rats; P1.hMR: *n* = 6 rats). To assess the prolonged effects of UVB irradiation on MGD on the cornea, a group of rats (WT: *n* = 6 rats; P1.hMR: *n* = 6 rats) were followed up until 2 weeks after UVB exposure. Corneal fluorescein staining was performed before, after 5 days of UVB and 2 weeks after UVB exposure. For assessment, the cornea was divided into four equal quadrants, and epithelial defects were graded in each quadrant using a 0–4 scoring system: 0 (no staining), 0.5 (minimal punctate staining), 1 (diffuse punctate staining), 2 (diffuse staining involving <1/3 of the area), 3 (diffuse staining involving >1/3 of the area), and 4 (staining involving >2/3 of the area)^70,71^. The final score was obtained by summing the scores of all four quadrants, and the scores from both eyes were averaged to obtain one value per animal for statistical analysis. Comparison was conducted between WT and P1.hMR at the timepoint 2 weeks post-UVB (*n* = 6 rats). Rats were then euthanized, eyelids and corneas were dissected for morphological and immunohistochemical analysis.

### Hematoxylin and eosin staining

Eyelid samples from humans and rats were immediately fixed in 10% neutral-buffered formalin for 24 h and 4% paraformaldehyde (PFA) for 2 h, respectively, followed by graded dehydration and embedding in paraffin. Serial sections of 7 µm thickness were obtained using a microtome (LEICA). The paraffin-embedded sections of human and rat eyelids were deparaffinized in xylene and rehydrated through a descending ethanol series. The sections were then stained with hematoxylin reagent (RAL Diagnostics, #320550-2500, Bordeaux, France) for 15 min and rinsed with tap water to remove excess dye. Subsequently, the sections were immersed in eosin solution (RAL Diagnostics, #312730-0100, Bordeaux, France) for 4 min and rinsed again with tap water. After sequential baths in absolute ethanol and xylene, the tissue sections were mounted using Eukitt® mounting medium (O. Kindler GmbH, #200080, Freiburg, Germany). Morphological changes were observed under a light microscope (Olympus BX51, Rungis, France) equipped with a CCD camera (Olympus DP70).

### Sirius red staining

For collagen detection, human and rat eyelid sections were deparaffinized and rehydrated, then incubated with 1% Sirius Red in picric acid for 15 min^72^. After sequential washes with acetic acid and absolute ethanol, the slides were mounted and examined under a microscope. Collagen content was quantified using Fiji ImageJ software (version 1.54b, Wayne Rasband, National Institutes of Health, Bethesda, MD, USA). A binary conversion of the red channel was performed to calculate the percentage of area occupied by collagen fibers. All quantifications were normalized to the analyzed tissue area.

### Immunohistochemistry

Paraffin-embedded rat and human eyelid sections were used for immunohistochemical staining. Briefly, sections were deparaffinized and rehydrated, followed by antigen retrieval in citrate buffer (10 mM, pH 6.0) at 100 °C for 20 min. Endogenous peroxidase activity was blocked using 3% hydrogen peroxide. Sections were then blocked with TNB blocking buffer (AKOYA Biosciences, #FP1012, Massachusetts, USA) for 30 min at room temperature. Primary antibodies (MR, GR, HSD1, HSD2, Nitrotyrosine, 4-HNE, 8OHgD, S100a9) were applied overnight at 4 °C as reference listed in Table. S3. After washes with PBST, sections were incubated with biotinylated secondary antibodies in Table. S3. for 1 h at room temperature. For GR and MR immunohistochemistry, the Tyramide Signal Amplification (TSA) biotin kit (AKOYA Biosciences, MA, USA) was used according to the manufacturer’s instructions to amplify the tyramide signal. For other primary antibodies, sections were processed with Vectastain Elite ABC reagent (Vector Laboratories, USA, #AK-5000) for 30 min. For S100A9, the signal was developed using the VIP Substrate Kit (Vector Laboratories, USA, #SK-4605) and stopped in PBS buffer. For other targets, the signal was visualized using 3,3’-diaminobenzidine (DAB) substrate (Vector Laboratories, USA, #SK-4105) and stopped in 50 mM TRIS buffer (pH 7.6) for 10 min. Tissue sections were mounted with Eukitt® mounting medium (O. Kindler GmbH, Freiburg, Germany)^41^. Negative controls were performed by omitting the primary antibody. For quantification, the positive staining area normalized to the same threshold for MR and GR, the mean grey value limited to the same threshold for staining intensity for other targets in MG were measured using ImageJ software (version 1.54b, National Institutes of Health, Bethesda, MD, USA).

### Quantitative PCR in ocular surface tissues from rats

RNA from various ocular surface tissues was isolated using the RNeasy Mini Kit (QIAGEN, Cat. No. 74106), followed by DNase I treatment (QIAGEN, Cat. No. 79254) according to the manufacturer’s protocol. Transcript levels were quantified by quantitative real-time PCR (qPCR) on the QuantStudio™ 5 Real-Time PCR system (Applied Biosystems, Foster City, CA, USA) using SYBR Green detection. Relative quantification was performed using the ΔΔCT method. Primer sequences for all analyzed genes are provided in Table. S4. Reference genes (*Hprt1*, *Ubc*, and *18S*) were used for normalization.

### Immunofluorescence

Rat ocular tissues were dissected, embedded in OCT compound, and frozen at −80 °C. Cryosections of 10 µm thickness were obtained using a cryostat (Leica). Immunofluorescence staining was performed. In brief, rat eyelid and corneal cryosections were fixed in 4% PFA followed by washes with PBS. Sections were permeabilized with 0.1% Triton X-100 in PBS for 30 min and blocked with 5% goat serum in PBS for 30 min at room temperature. Tissues were incubated with primary antibodies overnight at 4 °C. Secondary antibodies was applied the next day for 1 h at room temperature. Nuclei were counterstained with 4’,6-diamidino-2-phenylindole (DAPI; 1:5000) for 5 min, and slides were mounted using Gel Mount (Dako, Agilent, Les Ulis, France)^41^. Negative controls were performed by omitting the primary antibody. Sections were visualized using an Olympus fluorescence microscope (Olympus BX51). Primary and secondary antibodies, along with their dilutions, are listed in Table. S3. Quantification was performed using QuPath software.

### Nile red staining

Fresh-frozen rat MG sections were incubated with 1 µg/mL Nile red (Sigma-Aldrich, #72458, Missouri, USA) in PBS for 1 h at 4 °C. Tissues were then fixed in 4% paraformaldehyde (PFA) for 15 min, followed by DAPI staining and three washes with PBS. Stained sections were imaged using a confocal microscope (Zeiss LSM 710, Oberkochen, Germany). Strongly stained lipid droplets were quantified using Fiji ImageJ software by calculating the percentage of the highlighted area limited to threshold relative to the total analyzed area.

### RNA sequencing of rat MG and transcriptomic analysis

To characterize the pathology involved in P1.hMR phenotype, MGs were carefully dissected from 6-month-old WT (*n* = 6 eyes) and P1.hMR male rats (*n* = 4 eyes) for transcriptomic analysis. For UVB-induced MGD with ocular surface damages model of OR, 12-week-old Sprague-Dawley rats were irradiated by UVB on eyelids (WT: *n* = 6 eyes; P1.hMR: *n* = 10 eyes). Age- and sex-matched control rats (WT: *n* = 4; P1.hMR: *n* = 5) were raised in the same condition without model. Animals were euthanized after 5 days of UVB irradiation. RNA extraction was conducted as described above. 1 µg of the extracted RNA sample was sent for RNA sequencing at the iGenSeq transcriptomic platform of the Brain and Spine Institute (ICM, Paris, France). RNA quality was checked by capillary electrophoresis and RNA integrity numbers (RIN) ranging from 8.8 to 9.3 was accepted for library generation. Quality of raw data was assessed using FastQC. STAR v2.5.3 was used to align reads on reference genome rn7 using standard options. Between 30 and 38 million reads were mapped. Quantification of gene and isoform abundances was done with rsem 1.2.28, and reproducibility of replicates was controlled with PCA representations.

The transcriptomic data for rats were analyzed on the online platform of the Paris Brain Institute (quby.icm-institute.org). First, differential gene expression analysis with DESeq2 method was utilized to identify the differentially expressed genes. Adjusted P values (pFDR) were calculated with the Benjamini-Hochberg procedure to control false discovery rate (FDR threshold set at 0.05, log2 fold-change threshold set at 0.5). Then, an enrichment analysis was performed (by GSEA and over-representation analysis) in different gene-set collections including Reactome gene sets (Reactome subset of Canonical pathways), Hallmark gene sets (H), Gene Ontology gene sets (C5: GO) and Kyoto Encyclopedia of Genes and Genomes (KEGG) Pathway Gene Sets.

Transcriptomic data for human meibomian gland dysfunction (MGD) were reanalyzed using the GEO2R online platform (GSE17822), with differentially expressed genes (DEGs) identified based on a significance threshold of *P* < 0.05. For human rosacea, DEGs were obtained by comparing control samples with three different subtypes (logFC >1.5, *P* < 0.05) using GSE65914. An intersection analysis was performed to identify the most relevant DEGs across the three subtypes. Additional intersection analyses were conducted using the Xiantao Academic online platform (https://www.xiantaozi.com/).

### Statistics analysis

A study of the literature and an estimation using G-power software, version 3.1.9.6, were carried out to estimate the number of animals required. The investigators were blinded to group allocation during data collection and analysis. Data are expressed as mean ± SEM. Statistical analysis was performed using the GraphPad Prism (GraphPad Software, version 9, San Diego, CA, USA). The unpaired *t* test or Mann–Whitney test with two-sided comparisons was used to compare two groups. The Kruskal–Wallis test with Dunn’s multiple comparisons, or two-way ANOVA followed by Fisher’s LSD post hoc test or Tukey’s multiple comparisons, was used to compare more than two groups. A *P* value less than 0.05 was considered statistically significant.

### Reporting summary

Further information on research design is available in the Nature Portfolio Reporting Summary linked to this article.

## Supplementary information

Supplementary Information

Peer Review File

Description of Additional Supplementary Files

Supplementary Data 1

Reporting Summary

## Source data

Source Data

## Data Availability

All data supporting the findings described in the manuscript are available in the article, and in the Supplementary information and in the Source data file. Raw RNAseq data generated in this study have been deposited on Gene Expression Omnibus under accession code GSE291177. Other previously published data used in this study are publicly available under accession code GSE17822^28^; GSE65914^36^ and GSE245468^41^. Source data are provided with this paper.
