# Supplementary material for: Cross-species transcriptomics identify mineralocorticoid receptor pathway overactivation as a central driver of ocular rosacea
Source: Nat Commun. 2026 Apr 16;17:5247. doi: 10.1038/s41467-026-71945-4 (PMC13260418; doi:10.1038/s41467-026-71945-4)
Supplement: Supplementary file 1 — Supplementary Information [file 41467_2026_71945_MOESM1_ESM.pdf]

Figure S1

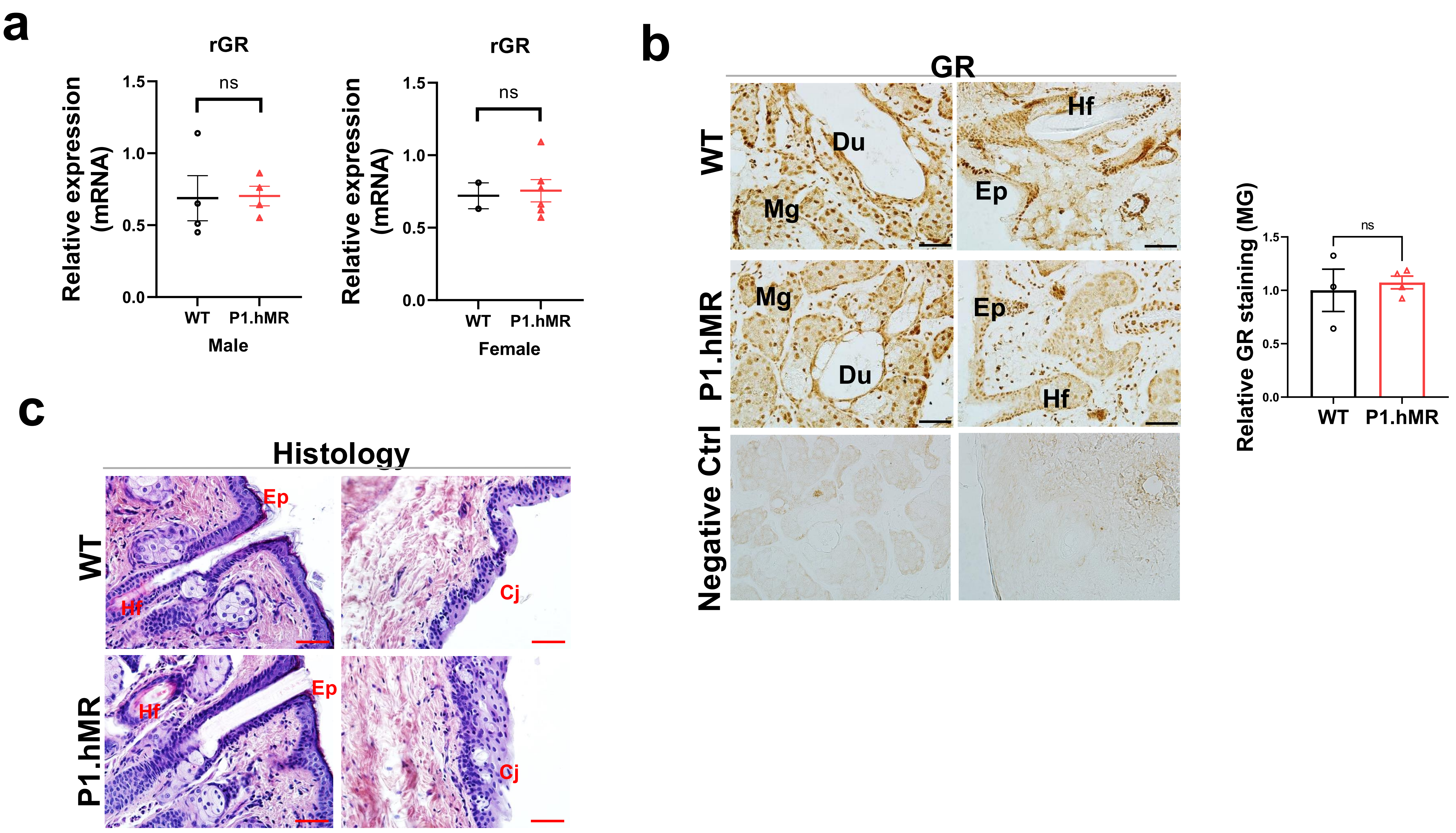

Fig. S1. hMR is overexpressed in the eyelid and ocular surface tissues of P1.hMR rats without altering GR expression

a. rGR expression in mRNA level in MG tissues of P1.hMR and WT rats. (n = 4 rats each group in Male; WT: n = 2 rats, P1.hMR: n = 6 rats in Female).

b. Immunohistochemistry of GR protein expression in ocular surface tissues of P1.hMR and WT rats. The protein level is determined by immunostaining and quantified by ImageJ (WT: n = 3 rats, P1.hMR: n = 4 rats).

c. Histology of the eyelid tissues including the epidemis, hair follicles and conjunctiva (n = 3-4 rats).

The specific locations of these tissues are indicated as below: Cj, conjunctiva; Mg, meibomian gland; Du, ducts; Ep, epidermis; Hf, hair follicles; CoEp, corneal epithelium. Scale bar: 100 um. Data expressed as mean ± SEM. ns, $p > 0.05$ (Mann-Whitney U test with two-sided comparison).

Figure S2

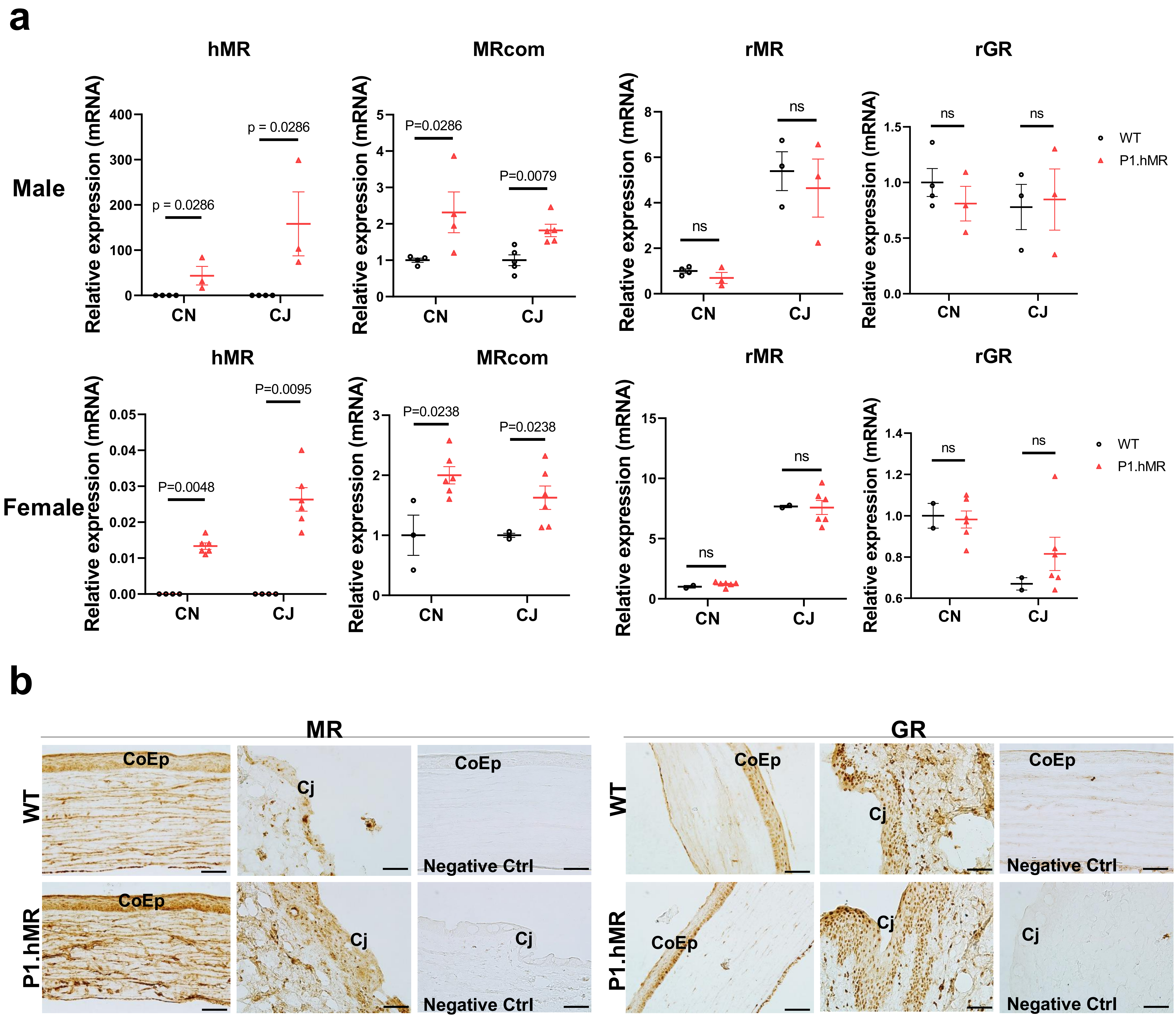

Fig. S2. hMR is overexpressed in the ocular surface tissues of P1.hMR rats without altering rMR and rGR expression

a. hMR, Mrcom, rMR and rGR expression in mRNA level in ocular surface tissues of P1.hMR and WT rats (For Male, WT: n = 4 rats, P1.hMR: n = 3 rats for hMR; n = 4 rats each group for CN and n = 5 rats each group for CJ for Mrcom; WT: n = 4 rats, P1.hMR: n = 3 rats for CN and n = 3 rats each group for CJ for rMR and rGR. For Female, WT: n = 4 rats, P1.hMR: n = 6 rats for hMR; WT: n = 3 rats, P1.hMR: n = 6 rats for Mrcom; WT: n = 2 rats, P1.hMR: n = 6 rats for rMR and rGR). CN, cornea; CJ, conjunctiva. Data expressed as mean ± SEM. ns, $p > 0.05$ (Mann-Whitney U test with two-sided comparison).

b. Immunohistochemistry of MR and GR protein expression in ocular surface tissues of P1.hMR and WT rats. (n = 3-4 rats). The specific locations of these tissues are indicated as below: Cj, conjunctiva; CoEp, corneal epithelium. Scale bar: 100 um.

Figure S3

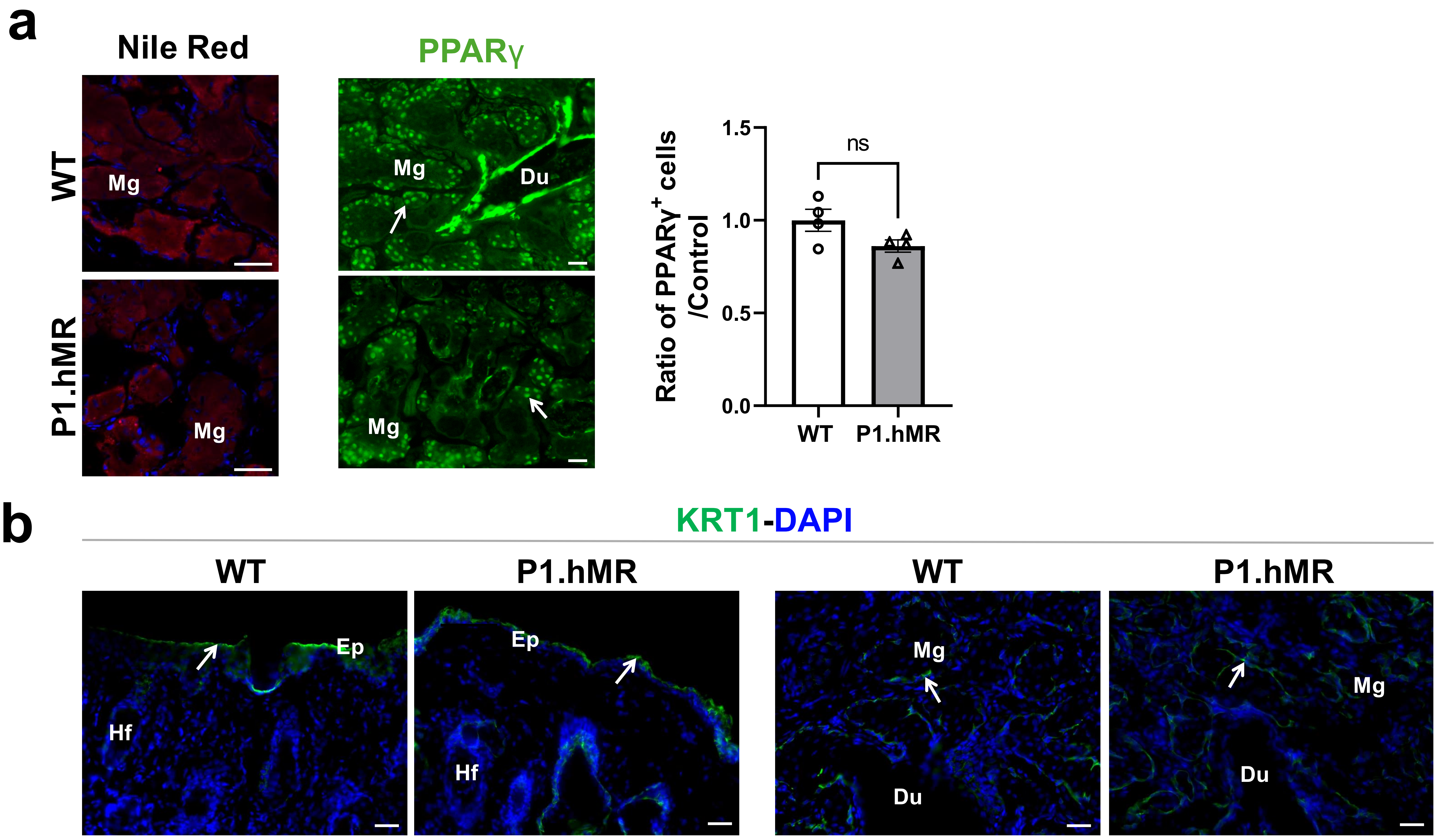

Fig. S3. No significant changes in lipid metabolism and keratinization between WT and P1.hMR rats.

a. Nile red staining (red color, left panel), right panel for lipogenesis and PPARγ protein expression in MGs from WT and P1.hMR rats. PPARγ protein level was determined by the ratio of positive immunosignal cells/ total DAPI-marked cells and quantified by QuPath (n = 4 rats).

b. KRT1 (green color) protein expression in the MGs from WT and P1.hMR rats (n = 4 rats).

The specific locations of these tissues are indicated as below: Mg, meibomian gland; Du, ducts; Ep, epidermis; Hf, hair follicles. Positive staining (arrows) are indicated. Scale bars: 100 μm. Data are presented as mean ± SEM; ns, $p > 0.05$ (Mann-Whitney U test with two-sided comparison).

Figure S4

Function	Genes	FC (Male)	P-value	FC (Female)	P-value
Immune Response and Inflammation	Cmklr1	1.61	0.01	1.38	0.02
	Itgb3	1.78	0.04	1.45	0.11
	Cfd	1.47	0.02	0.98	1.00
	Tlr3	1.01	0.60	1.33	0.01
	Nos3	1.66	0.03	1.21	0.47
	Cd36	1.16	0.40	1.48	0.02
	Mcp-1 (Ccl2)	1.19	0.22	1.23	0.04
	Clu	1.13	0.67	1.42	0.01
Cell Structure and extracellular matrix	Vim	1.20	0.51	1.40	0.04
	Pmel	1.83	0.01	1.65	0.03
	Col3a1	1.56	0.05	1.46	0.11
	Col1a1:	1.67	0.07	1.74	0.02
	Sdc4	1.34	0.00	1.20	0.12
	Fnln5	1.33	0.10	1.41	0.05
Metabolism and Energy Production	Cyp1b1	1.55	0.04	1.57	0.06
	Cyp1a1	1.55	0.04	1.63	0.04
	Pdha1	1.33	0.02	1.30	0.04
	Dld	1.23	0.02	1.15	0.38
	Crat	1.54	0.01	1.45	0.06
	Acsm3	1.37	0.02	1.17	0.46
Signal Transduction	Kcnj8	1.15	0.86	1.52	0.05
	Gas 1	1.13	0.52	1.50	＜0.0001
	Wipf1	1.49	0.04	1.26	0.47
	Stc1	1.67	0.00	1.29	0.31
Cell Adhesion	Tf	1.75	0.03	1.63	0.05
	Csrp1	1.29	0.01	1.29	0.01
Apoptosis and Cell Death	Aifm1	1.34	0.00	1.12	0.15

Fig. S4. Transcriptomic analysis of MGs tissues from P1.hMR versus WT rats.

Quantitative PCR confirmed the regulated genes by overexpressing hMR in MGs tissues that associated with MGD and OR pathologies. n = 3-6 rats in each group, precised n value and raw data were presented in the Source Data file. Significant fold change (FC) are highlighted (Mann-Whitney U test with two-sided comparison).

Figure S5

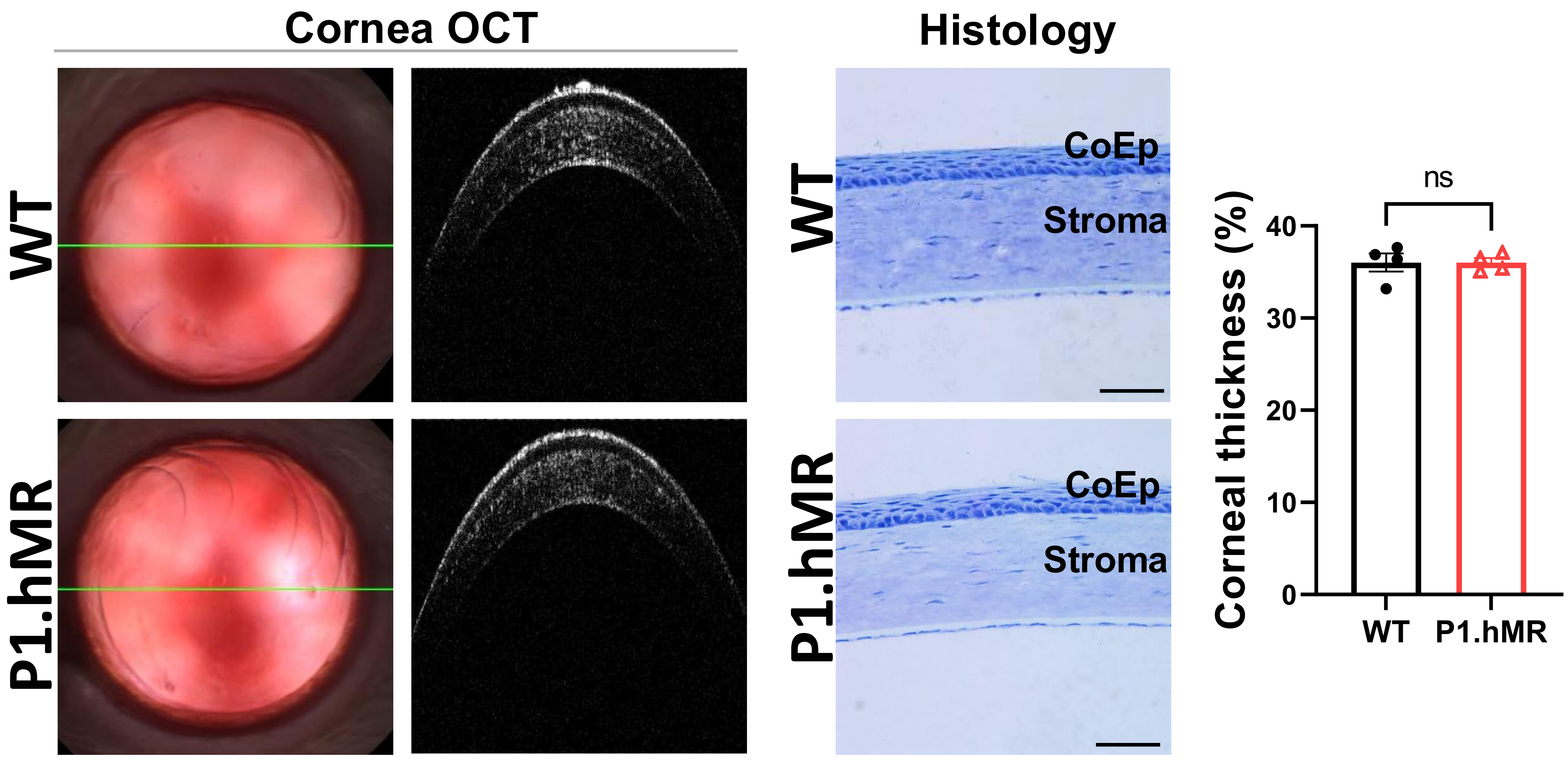

Fig. S5. Corneal Optical Coherence Tomography (OCT) imaging and histology show no significant differences between WT and P1.hMR rats overexpressing hMR.

Representative OCT images of cornea in WT and P1.hMR rats (Left panel). Histological sections showing corneal structure in WT and P1.hMR rats. Corneal thickness was quantified by ImageJ (n = 4 rats) (right panel).

The specific locations of these tissues are indicated as below: CoEp, corneal epithelium. Scale bar: 100 um. Data expressed as mean ± SEM. ns, $p > 0.05$ (Mann-Whitney U test with two-sided comparison).

Figure S6

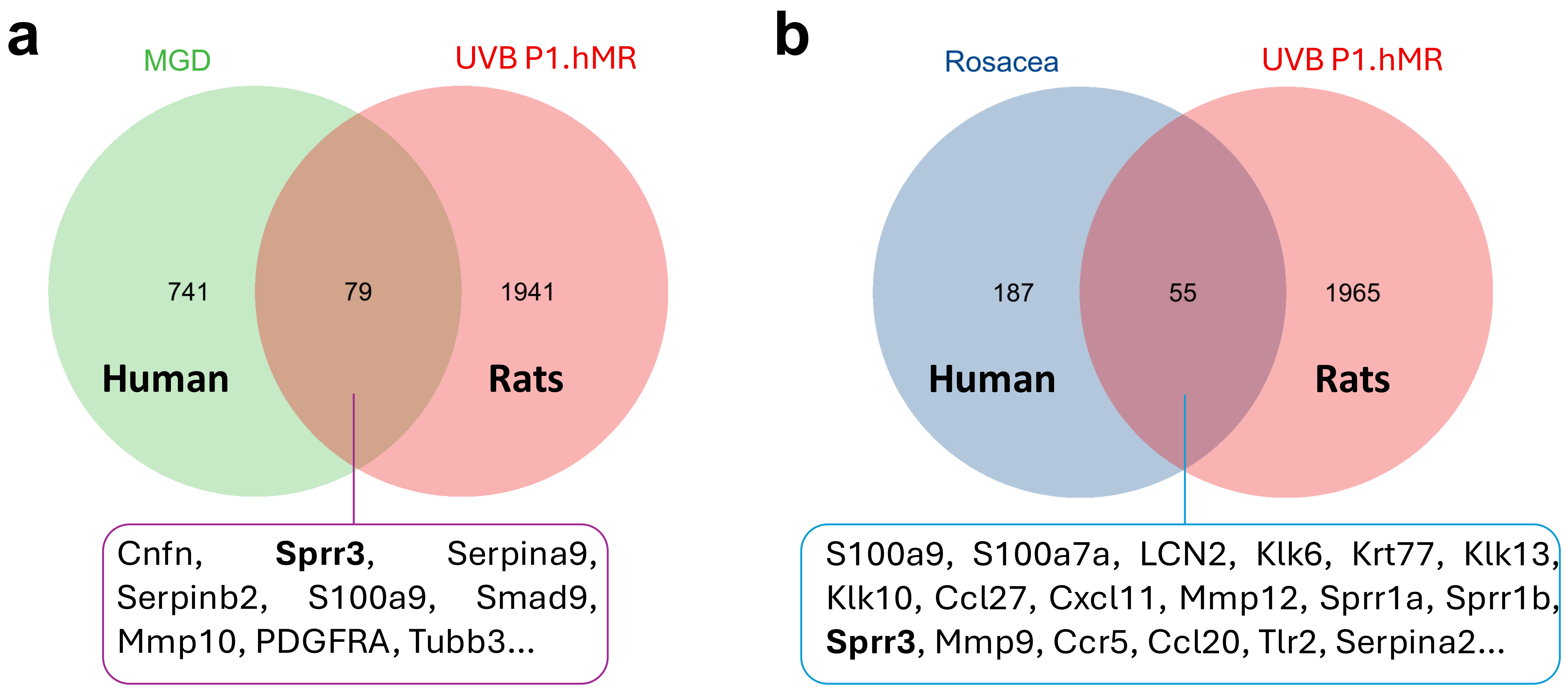

Fig. S6. Transcriptomic signature of UVB-induced pathology in MGs of P1.hMR rats shows significant similarities with MGs of human MGD and skin of human rosacea.

a. Venn diagrams illustrating overlap of differentially expressed genes (DEGs) between human MGD (GSE177822) and UVB-induced MGD in P1.hMR rats. DEGs significantly associated with MGD are listed.

b. Venn diagrams illustrating overlap of DEGs between human rosacea (GSE69514) and UVB-induced MGD in P1.hMR rats. DEGs significantly associated with rosacea are listed.

Figure S7

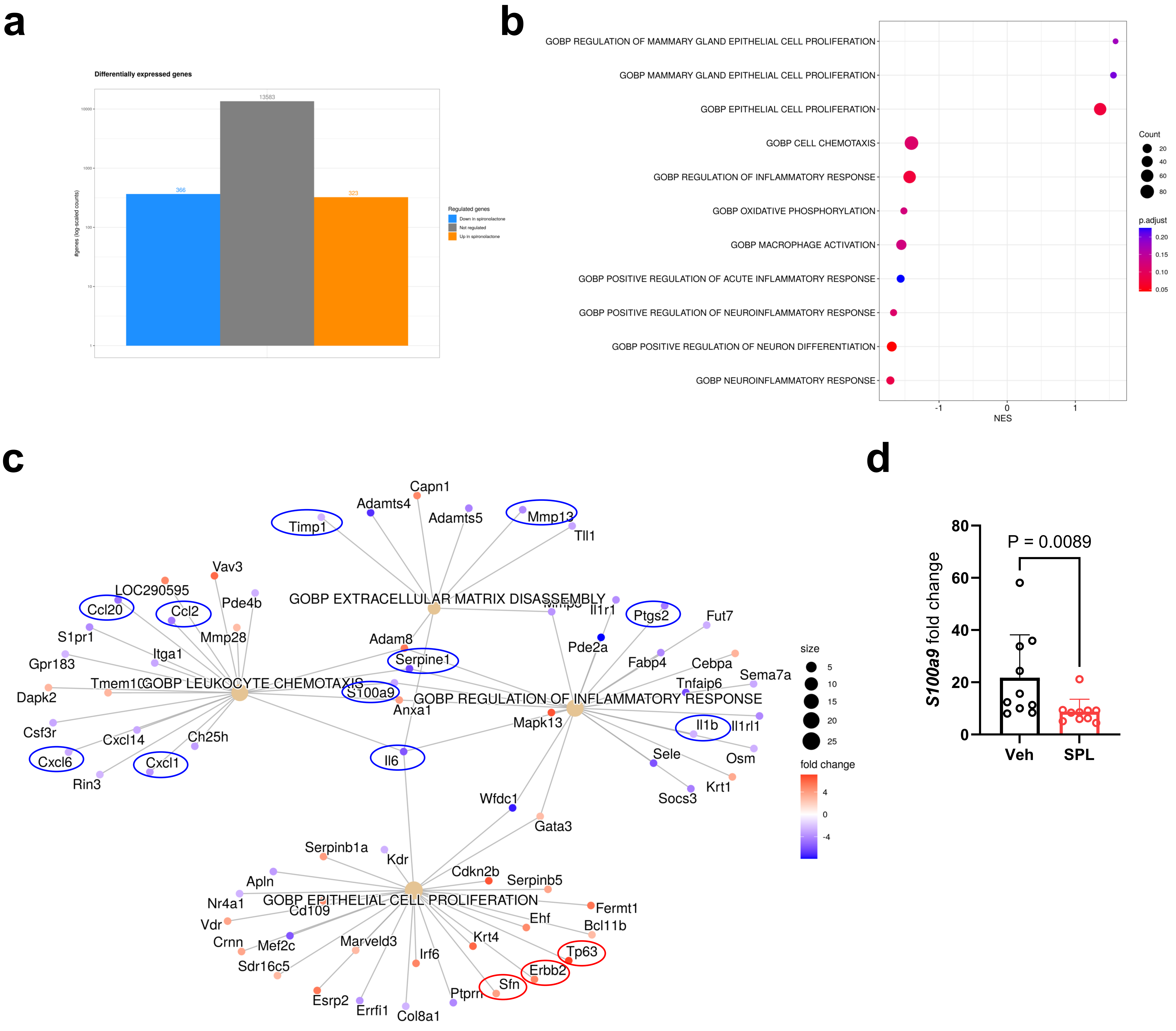

Fig. S7. Transcriptomic signature of cornea from Spironolactone treated rats vs. Vhichel group on day 3 after the LSCD model.

a. Differentially expressed genes regulated by Spironolactone treatment.

b. GSEA analysis using GOBP data set showing the significantly regulated pathways.

c. Gene concept network for significantly regulated genes by spironolactone in the rat cornea at day 3 after LSCD model identified genes related to leukocyte chemotaxis, inflammatory response, extracellular matrix disassembly and epithelial cell proliferation.

d. PCR analysis validated the downregulation of S100a9 by spironolactone treatment.

Raw RNAseq data is available on Gene Expression Omnibus under accession code GSE245468 [https://www.ncbi.nlm.nih.gov/geo/query/acc.cgi?acc=GSE245468] .

Antibodies used in the paper and corresponding negative control.

Rabbit anti-P63α (1:100, Cell Signaling Technology, #4892, Ozyme, Saint-Cry-L'Ecole, France);

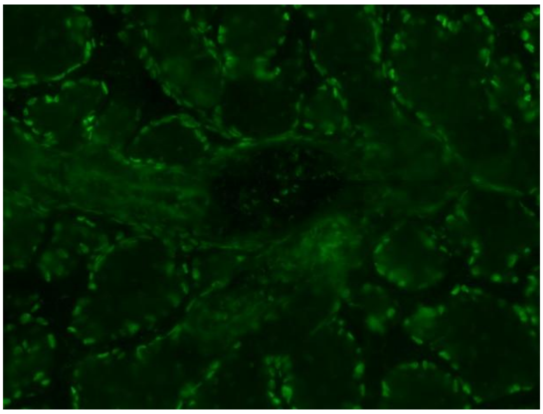
Positive staining

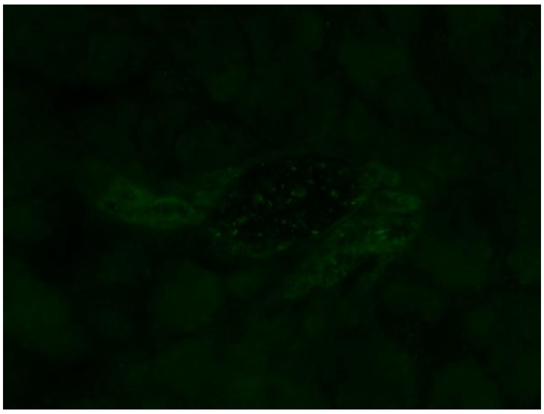
Negative control

rabbit anti-PPARγ (1:100, Cell Signaling Technology, #2435S, Massachusetts, USA);

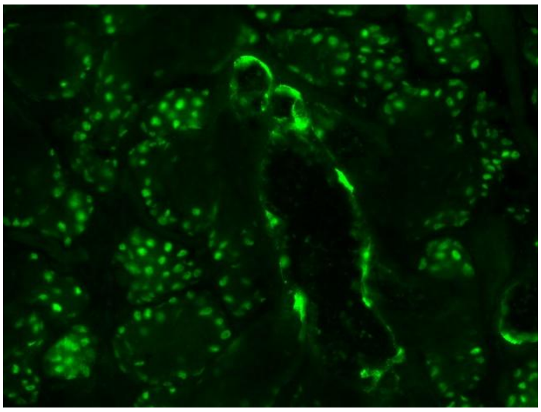
Positive staining

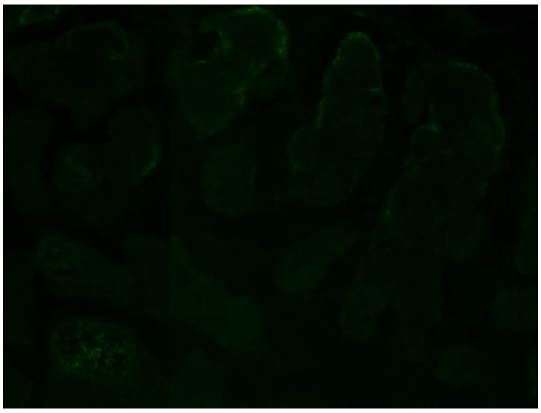
Negative control

mouse anti Ki67 (1:100, Cell Signaling Technology, #9449 (8D5), Massachusetts, USA);

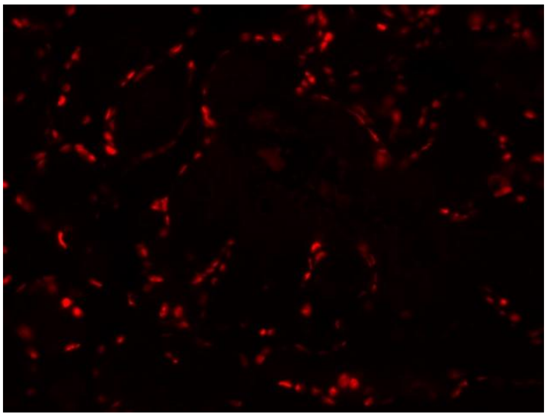
Positive staining

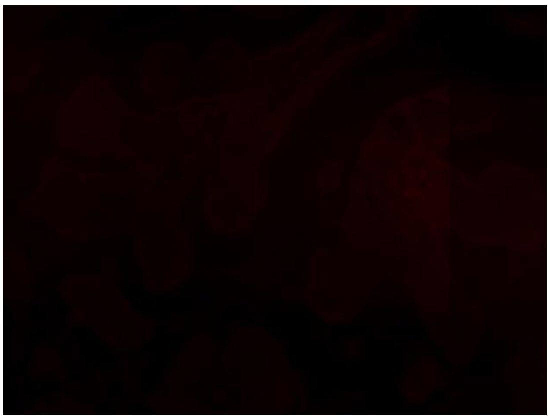
Negative control

rabbit anti Cytokeratin 1 (1:500, Abcam, #ab185628);

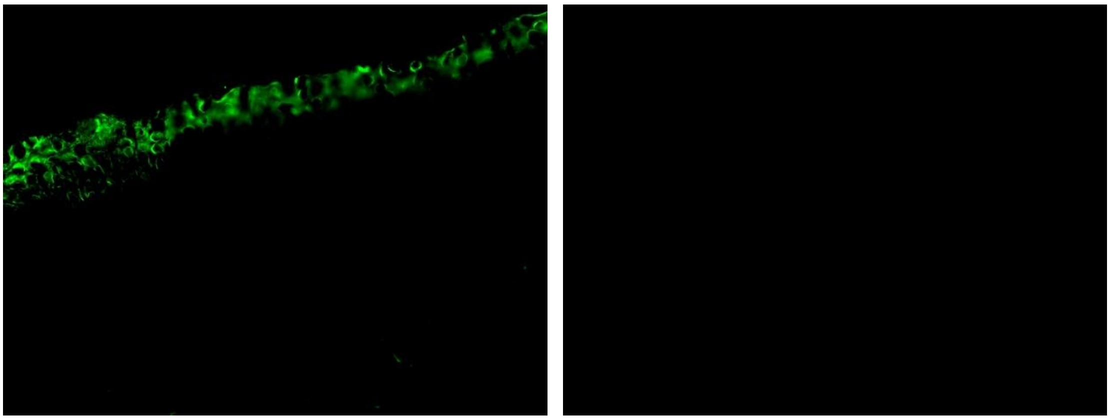

Positive staining Negative control

rabbit anti Cytokeratin 10 (1:500, Abcam, #ab76318) in red A597;

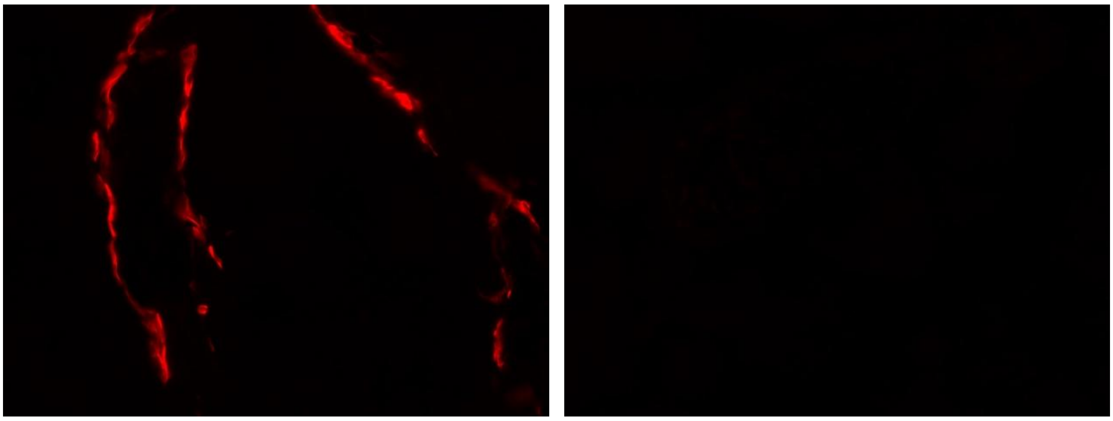

Positive staining Negative control

rabbit anti-IBA1 (1:400, Wako #019-19741, Richmond, VA, USA);

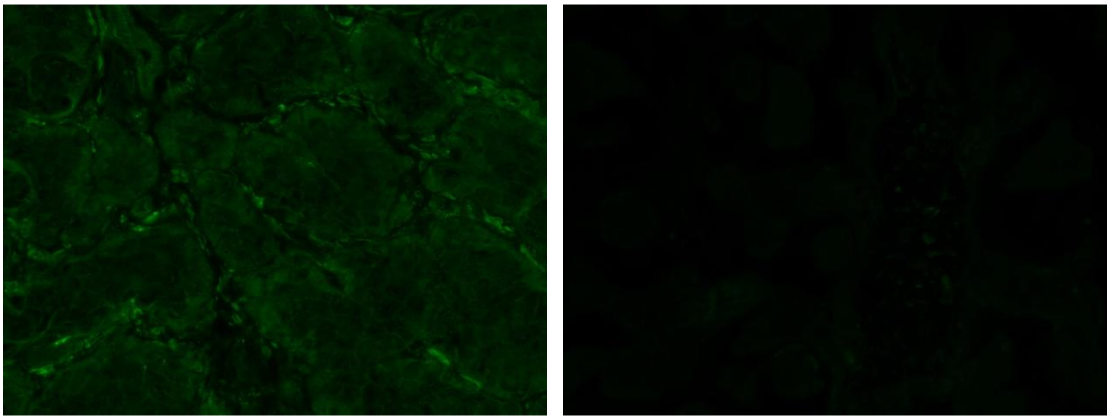

Positive staining Negative control

mouse anti-ED1 (1:200, Bio-Rad #MCA341R, Colmar, France);

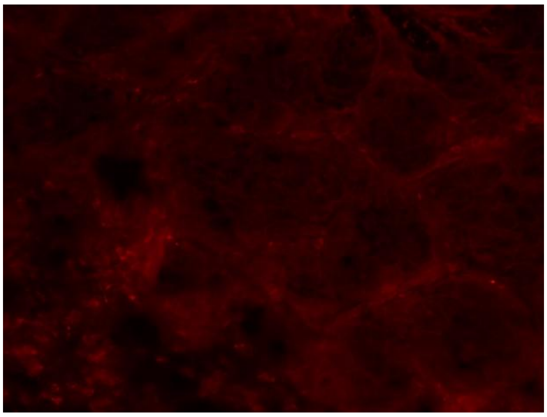

Positive staining

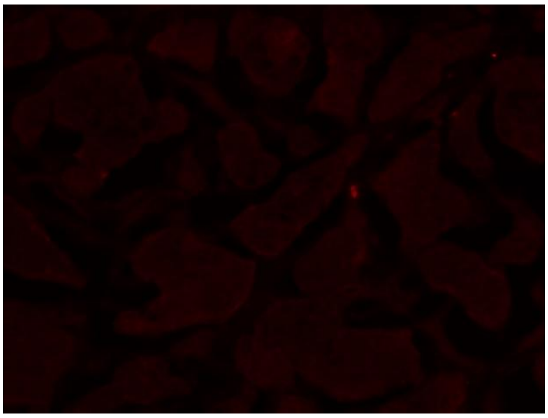

Negative control

rabbit anti TIM23 (1:100, Proteintech #11123-1-AP, Planegg-Martinsried, Germany);

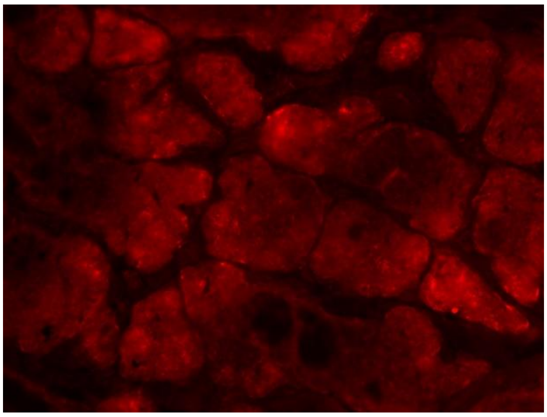

Positive staining

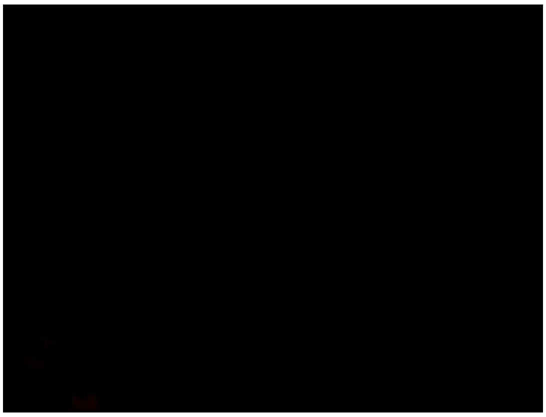

Negative control

rabbit anti TOM20 (1:400, Proteintech #11802-1-AP);

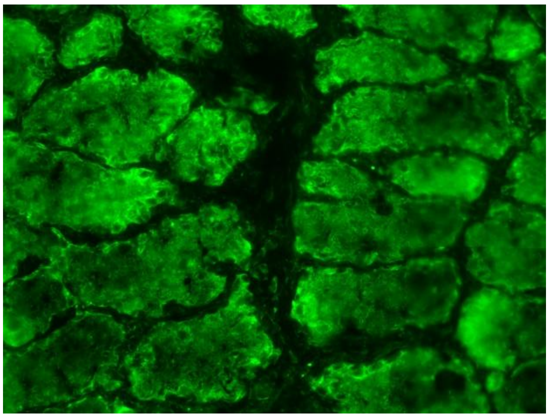

Positive staining

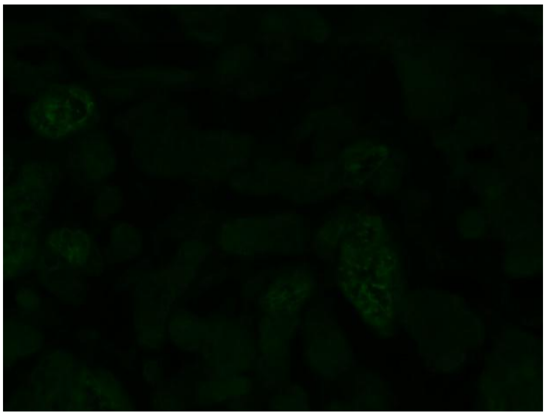

Negative control

mouse anti E-Cadherin (1:400, Abcam #ab231303);

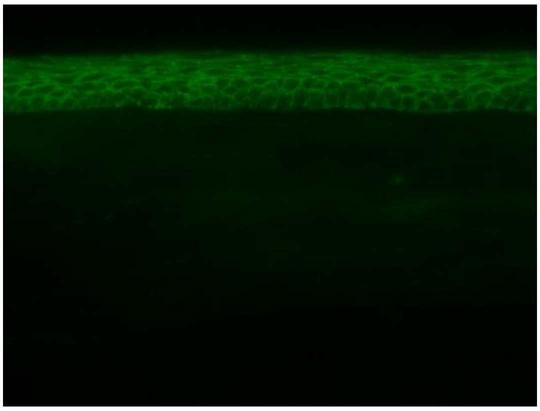

Positive staining

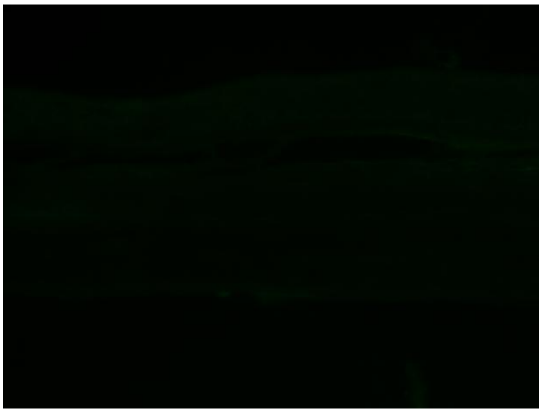

Negative control

rabbit anti ZO-1 (1:200, Thermo Fisher Scientific, #40-2200,);

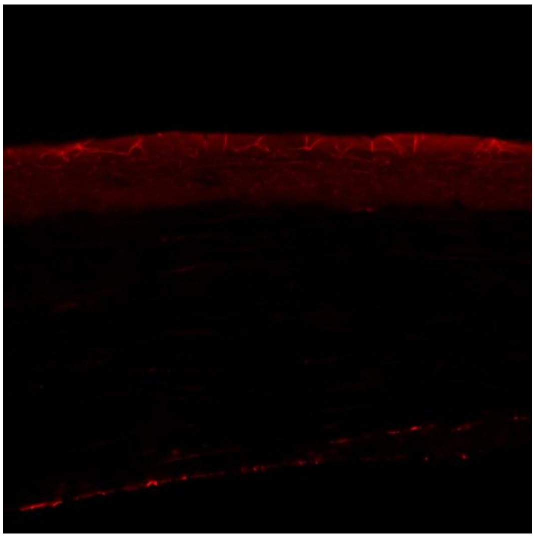

Positive staining

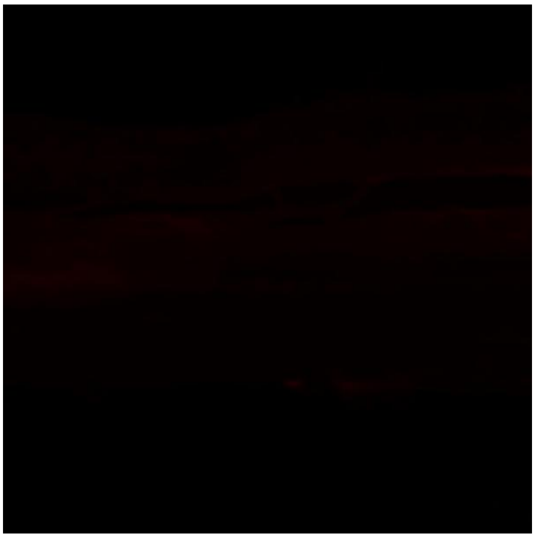

Negative control

Table. S1_Commonly regulated genes in the P1h.MR rat UVB-induced MGD and OR model and in MGD patients

MGD AND UVB P1hMR		
ABAT	ADH7	ACOT2
ADORA2B	AFF3	ALDH3A1
APOBEC2	AURKA	BNC1
CCSER1	CDC14B	CELF4
CENPE	CHRNA1	CNFN
CROCC	CYSRT1	DACT1
DLC1	DTL	ECE2
ECT2	FAM72A	FCGBP
FKBP10	GALNS	GCNT2
GRHL3	GSDMC	HSPB7
IL18BP	JAKMIP1	KANK4
KCNH2	KERA	KPNA2
KRT18	LCE1F	LDHC
LRTM2	LYNX1	MAL
MCC	MGST2	MME
MMP10	MYF6	NTRK3
OIP5	ORC1	PDGFRA
PLXNC1	PMAIP1	PPP1R3A
PSD2	PVALB	RAVER2
RCAN2	RELN	RET
S100A9	SERPINA9	SERPINB2
SKA3	SLC27A6	SLC39A8
SLURP1	SMAD9	SPRR3
TMPRSS11A	TMPRSS11B	TMPRSS11D
TPPP	TPX2	TUBB3
WASF3	WDR60	ZBTB20
ZBTB4		

Table. S2_Commonly regulated genes in the P1h.MR rat UVB-induced MGD and OR model and in skin of human rosacea

Rosacea AND UVB P1hMR		
S100A7A	S100A9	LCN2
WDR72	KLK6	ATP12A
SPP1	SLCO4C1	KRT77
CCL27	PCOLCE2	PCP4
VNN1	CXCL11	PCP4L1
MMP12	LIPG	PEG3
KLK13	SCARA5	SPRR1A
PLLP	SPRR1B	ARG2
CHI3L1	SLC14A1	CDHR1
CD2	ID4	FBP1
XK	SPRR3	MMP9
CRIP1	SLC12A8	GATA6
GJB2	ADAMTSL3	CCR5
CCL20	C1QB	ACPP
TLR2	PTGDS	SLC51A
SLC6A14	SERPINA12	CMAHP
PTPN21	ADRB2	AREG
ADH6	GIPC2	SLC38A4
KLK10		

Table. S3 List of antibodies used in immunofluorescence study

Antibodies	Dilution	Reference
Primary antibodies		
rabbit anti-P63α	1:100	Cell Signaling Technology, #4892, Ozyme, Saint-Cry-L'Ecole, France
mouse anti Acrolein	1:100	Abcam, #ab240918
8-OHdG/8 Hydroxyguanosine rabbit Antibody	1:100	Thermo Fisher Scientific, BS-1278R
rabbit anti-PPARγ	1:100	Cell Signaling Technology, #2435S
mouse anti Ki67	1:100	Cell Signaling Technology, #9449 (8D5), Massachusetts, USA
rabbit anti Cytokeratin 1	1:500	Abcam, #ab185628
rabbit anti Cytokeratin 10	1:500	Abcam, #ab76318
rabbit anti Cytokeratin 14	1:500	Abcam, #ab181595
rabbit anti-IBA1	1:400	Wako #019-19741, Richmond, VA, USA
mouse anti-ED1	1:200	Bio-Rad #MCA341R, Colmar, France
rabbit anti TIM23	1:100	Proteintech #11123-1-AP, Planegg-Martinsried, Germany
rabbit anti TOM20	1:400	Proteintech #11802-1-AP
mouse anti E-Cadherin	1:400	Abcam #ab231303
rabbit anti ZO-1	1:200	Thermo Fisher Scientific, #40-2200
mouse monoclonal anti-MR 6G1	1:2000	Merck, #MABS496, Darmstadt, Germany
rabbit anti-GR	1:1500	Santa Cruz Biotechnology, #sc-393232, Texas, USA
mouse anti-11β-HSD1	1:500	Santa Cruz Biotechnology, Texas, USA
mouse anti-11β-HSD2	1:500	Santa Cruz Biotechnology, Texas, USA
rabbit anti-Nitrotyrosine	1:200	Thermo Fisher Scientific, #BS-8551R, Saint Aubin, France
rabbit anti-4-Hydroxynonenal (4-HNE)	1:200	Abcam, ab46545, Cambridge, UK

rabbit anti-S100A9	1:300	Abcam, ab63818
Secondary antibodies		
Alexa Fluor 488-conjugated goat anti-rabbit IgG	1:200	Thermo Fisher Scientific, #A11008
Alexa Fluor, Alexa Fluor 488-conjugated donkey anti-mouse IgG	1:200	Thermo Fisher Scientific, # A-21202
Alexa Fluor 594-conjugated goat anti-rabbit IgG	1:200	Thermo Fischer Scientific, #A11012
Alexa Fluor 594-conjugated donkey anti-mouse IgG	1:200	Thermo Fischer Scientific, #21203
biotinylated goat anti-mouse secondary antibody	1:500	Vector Laboratories, #BA-2000, Eurobio Scientific, Les Ulis, France
biotinylated goat anti-rabbit secondary antibody	1:500	Vector Laboratories, #BA-1000, Eurobio Scientific, Les Ulis, France

Table. S4 List of primers used in this study

Gene Name	Forward/Reverse	Sequences
18S	Forward	TGCAATTATTCCCCATGAACG
18S	Reverse	GCTTATGACCCGCACTTACTGG
Ubc	Forward	ATCTAGAAAGAGCCCTTCTTGTGC
Ubc	Reverse	ACACCTCCCCATCAAACCC
Hprt1	Forward	GCGAAAGTGGAAAAGCCAAGT
Hprt1	Reverse	GCCACATCAACAGGACTCTTGTAG
Nr3c2 (human)	Forward	CCCTCTGAACATGACATCTTCG
Nr3c2 (human)	Reverse	CTGGAGCCTCGATTTTCAAC
Nr3c2 (rat)	Forward	TAAGTTTCCCCACGTGGTTC
Nr3c2 (rat)	Reverse	ATCCACGTCTCATGGCTTTC
NR3C1 (rat)	Forward	AAC ATG TTA GGT GGG CGT CAA
NR3C1 (rat)	Reverse	GGT GTA AGT TTC TCA AGC CTA GTA TCG
Cmklr1	Forward	TATTCTGCAACAGTGAACAG
Cmklr1	Reverse	AAGCTGTAGATTACCACCAG
Itgb3	Forward	AAAAACCCCTGTTACACTATG
Itgb3	Reverse	TTCACTTCGTCATTGAAGC
Cfd	Forward	CAAGCATTTGTATGATGTGC
Cfd	Reverse	TTGTGGGAGAGCTTAAAGAG

Gene Name	Forward/Reverse	Sequences
Tlr3	Forward	CTT AAA GAG TTT TCT CCA GGG
Tlr3	Reverse	ATT CTG GAT GCT TGT GTT TG
Nos3	Forward	CAC CGA TAC AAC ATA CTT GAG
Nos3	Reverse	CCT TCT GCT CAT TTT CCA AG
Cd36	Forward	AAG GAA TTT GTC CTA TTG GG
Cd36	Reverse	GAG ACT TCT CAA CAA AAG GTG
Mcp-1	Forward	TTC CTT ATT GGG GTC AGC AC
Mcp-1	Reverse	CAG TTA ATG CCC CAC TCA CC
Clu	Forward	AGC ACA TAA AGA CCC TCA TAG
Clu	Reverse	TCA GCT TCA TTT CAG AAT CC
Vim	Forward	CAGAACATGAAGGAAGAGATG
Vim	Reverse	TCCAGGTTAGTTTCTCTCAG
Pmel	Forward	TATAGAAGGATCCAGGAATCAG
Pmel	Reverse	ATGGAAAAGGAGGTATTTGC
Col3a1	Forward	TTT CAA GAT CAA CAC TGA GG
Col3a1	Reverse	TAT TCT CCG CTC TTG AGT TC
Col1a1	Forward	GCC TCC CAG AAC ATC ACC TA
Col1a1	Reverse	ATG TCT GTC TTG CCC CAA GT
Sdc4	Forward	GAGATCTAGATGACACGGAG
Sdc4	Reverse	CATTCTCTTCCAGTTCCTTG

Gene Name	Forward/Reverse	Sequences
Fnln5	Forward	GTCAACATGAGTGTGTGAAC
Fnln5	Reverse	CACATTCATTGATATCCTGGC
Cyp1b1	Forward	CCACTATTACAGACATCTTTGG
Cyp1b1	Reverse	CATGACGTATGGTAAGTTGG
Cyp1a1	Forward	AACCATGACCAGGAACTATG
Cyp1a1	Reverse	AGAATGACCTTCTCACTCAG
Pdha1	Forward	CGTAATTTCGCAAATGATGC
Pdha1	Reverse	TCTGCTTATACAGCTGATCC
Dld	Forward	CAAGAATCAGGTTACAGCTAC
Dld	Reverse	CTTCATCAATCGTGATTCCG
Crat	Forward	GGAGTATACAAAGAAGCCTG
Crat	Reverse	GAAGTCCTTTCCAAAGTGATG
Acsm3	Forward	CATCACTGTCTTCTGTTCTG
Acsm3	Reverse	TCTGTCTGTCCATATCCTTC
Kcnj8	Forward	AATGTCAGGTCATTCACTTC
Kcnj8	Reverse	CTCAGTCATCATTCTCCCTC
Gas1	Forward	CCCCGTTTGCCTGTTTTCTG
Gas1	Reverse	CAGTGCTCCGATCATCTCC
Wipf1	Forward	TCTACTTCCATCCGATTTCC
Wipf1	Reverse	TCTTCTGTTGGATCCACTTC

**Gene Name**	**Forward/Reverse**	**Sequences**
Stc1	Forward	TGT TCT ACT TTC CAG AGG ATG
Stc1	Reverse	TCT GTT GTA GTA TCT GTT GGA G
Tf	Forward	AACCATATTTGAGGTCTTGC
Tf	Reverse	CTCTTTGCCATCTCCATTTC
Csrp1	Forward	CATCCAAGTTTGCTCAGAAG
Csrp1	Reverse	CCTTTGCAATAGATCTCACC
Aifm1	Forward	CAAGAAGTCTGTCTGCTATC
Aifm1	Reverse	GTAATTGACTTGACTTCCCG
