# Supplementary material for: Cross-species transcriptomics identify mineralocorticoid receptor pathway overactivation as a central driver of ocular rosacea
Source: Nat Commun. 2026 Apr 16;17:5247. doi: 10.1038/s41467-026-71945-4 (PMC13260418; doi:10.1038/s41467-026-71945-4)
Supplement: Supplementary file 3 — Description of Additional Supplementary Files [file 41467_2026_71945_MOESM3_ESM.pdf]

Description of Additional Supplementary Files

Supplementary Data 1 : Differentially expressed (DE) genes in the rat cornea treated with SPL vs vehicle in LSCD (limbal stem cell deficiency) model
LogFC: log fold change, FC : fold change, FDR: false discovery rate
