# Supplementary material for: Cross-species transcriptomics identify mineralocorticoid receptor pathway overactivation as a central driver of ocular rosacea
Source: Nat Commun. 2026 Apr 16;17:5247. doi: 10.1038/s41467-026-71945-4 (PMC13260418; doi:10.1038/s41467-026-71945-4)
Supplement: Supplementary file 5 — Reporting Summary [file 41467_2026_71945_MOESM5_ESM.pdf]

nature portfolio

Corresponding author(s): Francine BEHAR-COHEN

Last updated by author(s): 2026/01/16

Reporting Summary

Nature Portfolio wishes to improve the reproducibility of the work that we publish. This form provides structure for consistency and transparency in reporting. For further information on Nature Portfolio policies, see our Editorial Policies and the Editorial Policy Checklist.

Statistics

For all statistical analyses, confirm that the following items are present in the figure legend, table legend, main text, or Methods section.

n/a	Confirmed	
☐	☒	The exact sample size (*n*) for each experimental group/condition, given as a discrete number and unit of measurement
☐	☒	A statement on whether measurements were taken from distinct samples or whether the same sample was measured repeatedly
☐	☒	The statistical test(s) used AND whether they are one- or two-sided<br>*Only common tests should be described solely by name; describe more complex techniques in the Methods section.*
☐	☒	A description of all covariates tested
☐	☒	A description of any assumptions or corrections, such as tests of normality and adjustment for multiple comparisons
☐	☒	A full description of the statistical parameters including central tendency (e.g. means) or other basic estimates (e.g. regression coefficient) AND variation (e.g. standard deviation) or associated estimates of uncertainty (e.g. confidence intervals)
☐	☒	For null hypothesis testing, the test statistic (e.g. *F*, *t*, *r*) with confidence intervals, effect sizes, degrees of freedom and *P* value noted<br>*Give P values as exact values whenever suitable.*
☒	☐	For Bayesian analysis, information on the choice of priors and Markov chain Monte Carlo settings
☒	☐	For hierarchical and complex designs, identification of the appropriate level for tests and full reporting of outcomes
☒	☐	Estimates of effect sizes (e.g. Cohen's *d*, Pearson's *r*), indicating how they were calculated

Our web collection on statistics for biologists contains articles on many of the points above.

Software and code

Policy information about availability of computer code

Data collection	Zeiss ZEN Software (commercial): Version 3.6 (blue edition) was utilized to acquire and process microscopic images.
Data analysis	QuPath (open-source): Version 0.4.3 was used for histological image analysis and annotation of tissue samples.GraphPad Prism (commercial): Version 9.5.1 was employed for statistical analyses, including regression and group comparisons.Zeiss ZEN Software (commercial): Version 3.6 (blue edition) was utilized to acquire and process microscopic images.Microsoft Excel (commercial): Version 2021 was used for data organization and initial calculations. Fiji (open-source): Version 2.1.0 was used for image analysis and processing.

For manuscripts utilizing custom algorithms or software that are central to the research but not yet described in published literature, software must be made available to editors and reviewers. We strongly encourage code deposition in a community repository (e.g. GitHub). See the Nature Portfolio guidelines for submitting code & software for further information.

Data

Policy information about availability of data

All manuscripts must include a data availability statement. This statement should provide the following information, where applicable:

- Accession codes, unique identifiers, or web links for publicly available datasets
- A description of any restrictions on data availability
- For clinical datasets or third party data, please ensure that the statement adheres to our policy

All Data are available upon reasonable request. Raw RNAseq data generated in this study have been deposited on Gene Expression Omnibus under accession code

GSE291177 [https://www.ncbi.nlm.nih.gov/geo/query/acc.cgi?acc=GSE291177]. Other previously published data used in this study are publicly available under accession code GSE17822 [https://www.ncbi.nlm.nih.gov/geo/query/acc.cgi?acc=GSE17822];GSE65914[https://www.ncbi.nlm.nih.gov/geo/query/acc.cgi?acc=GSE245468] and GSE245468 [https://www.ncbi.nlm. nih.gov/ geo/query/acc.cgi?acc=GSE245468]. Source data are provided with this paper.

Research involving human participants, their data, or biological material

Policy information about studies with human participants or human data. See also policy information about sex, gender (identity/presentation), and sexual orientation and race, ethnicity and racism.

Reporting on sex and gender	Human eyelid donors of both biological sexes (male and female) were included, with 3 male and 1 female in OR patients and control subjects. Sex was determined based on clinical records at the time of tissue collection; gender identity was not collected. Age and underlying ocular or systemic diseases were considered in the study design and analysis. The findings are applicable to both sexes, and sex-based comparisons were performed where appropriate; no significant sex-specific differences were observed unless otherwise stated. All participants provided written informed consent for the use of their clinical data and tissues for research purposes, in accordance with institutional and ethical guidelines.
Reporting on race, ethnicity, or other socially relevant groupings	No socially constructed or socially relevant categorization variables (such as race, ethnicity, socioeconomic status, or gender identity) were collected or used in this study. The only participant characteristic recorded was biological sex, which was obtained from clinical or donor records at the time of tissue collection and used solely as a biological variable rather than a social construct. These socially constructed variables were not collected because the tissues were obtained from a biobank with de-identified donor information, and such data were not available or required for addressing the biological aims of the study.<br>Classification into categories such as race, ethnicity, or gender identity was not performed, and no proxy variables were used in place of these factors.<br>No analyses based on socially constructed groupings were performed.
Population characteristics	See above.
Recruitment	OR patients and control subjects were diagnosed by both an ophthalmologist and a dermatologist to ensure the ocular signs and rosacea. Inflammatory conditions other than OR were excluded. Resection surgeries were performed due to the diagnosis of ectropion, entropion or plastic surgery (blepharoplasty surgery). The surgical pieces were immediately fixed in 10% formalin for 24 hours, dehydrated, embedded in paraffin wax and cut into 7 um thick slices and stained with hemalum-eosine, for histological analysis. Diagnosis of OR patients and control subjects were further confirmed by a pathologist on the sections.
Ethics oversight	The collection and storage of human biological samples were approved by local ethics committee CCP Ile de France 1 (no. 2016-nov-14390). All patients signed written consent for non-opposition to the use of surgical waste in research in line with Article L1211-2 of the French Public Health Code on biological samples collected during routine clinical care, reused for research purposes.

Note that full information on the approval of the study protocol must also be provided in the manuscript.

Field-specific reporting

Please select the one below that is the best fit for your research. If you are not sure, read the appropriate sections before making your selection.

☒ Life sciences ☐ Behavioural & social sciences ☐ Ecological, evolutionary & environmental sciences

For a reference copy of the document with all sections, see nature.com/documents/nr-reporting-summary-flat.pdf

Life sciences study design

All studies must disclose on these points even when the disclosure is negative.

Sample size	For all animal study, either only one eye was analyzed or when both eyes were analyzed they were polled and considered as one single sample, at least 4 to 5 animals were used for each analysis and the experiments were reproduced at least twice. This number has been estimated based on the models that were developped and their reproducibility and chosen to limit the number of animals sacrificed to agree with the 3R in animal for research.
Data exclusions	We did not exclude data unless statistical analysis identified these data as outlier
Replication	We have reproduced the experiments at least two times and the animal model that was used in this paper UV-induced OR has been recently published in IOVS with full descriptions and validations
Randomization	Animals were randomized when exposed to UV-B.
Blinding	The clinical scoring was performed by an ophthalmologist in a masked manner.

Reporting for specific materials, systems and methods

We require information from authors about some types of materials, experimental systems and methods used in many studies. Here, indicate whether each material, system or method listed is relevant to your study. If you are not sure if a list item applies to your research, read the appropriate section before selecting a response.

Materials & experimental systems

n/a	Involved in the study
☐	☒ Antibodies
☒	☐ Eukaryotic cell lines
☒	☐ Palaeontology and archaeology
☐	☒ Animals and other organisms
☐	☒ Clinical data
☒	☐ Dual use research of concern
☒	☐ Plants

Methods

n/a	Involved in the study
☒	☐ ChIP-seq
☒	☐ Flow cytometry
☒	☐ MRI-based neuroimaging

Antibodies

Antibodies used

Primary antibodies:
rabbit anti-Nitrotyrosine Thermo Fisher Scientific #BS-8551R Rat Rabbit / IgG 1/200 Saint Aubin, France
rabbit anti-P63α Cell Signaling Technology #4892 Human, Rat Rabbit 1/100 Massachusetts, USA
rabbit anti-PPARγ Cell Signaling Technology #2435S Human, Rat, mouse Rabbit IgG 1/100 Massachusetts, USA
mouse anti Ki67 Cell Signaling Technology #9449 Human, Rat, mouse Mouse IgG1 1/100 Massachusetts, USA
rabbit anti Cytokeratin 1 Abcam #ab185628 Human, Rat, mouse Rabbit 1/500 Cambridge, United Kingdom
rabbit anti Cytokeratin 10 Abcam #ab76318 Human, Rat, mouse Rabbit 1/500 Cambridge, United Kingdom
mouse anti-ED1 Bio-Rad #MCA341R Human, Rat, mouse Mouse IgG1 1/200 Colmar, France
rabbit anti TIM23 Proteintech #11123-1-AP human, mouse, rat Rabbit / IgG 1/100 Planegg-Martinsried, Germany
rabbit anti TOM20 Proteintech #11802-1-AP human, mouse, rat Rabbit / IgG 1/100 Planegg-Martinsried, Germany
mouse anti E-Cadherin Abcam #ab231303 Human, Mouse, Rat mouse 1/400 Cambridge, United Kingdom
rabbit anti ZO-1 Thermo Fisher Scientific #40-2200 Human, Mouse, Rat Rabbit / IgG 1/200 Saint Aubin, France
mouse anti Acrolein Abcam #ab240918 Human, Mouse, Rat Mouse 1/100 Cambridge, United Kingdom
8-OHdG/8 Hydroxyguanosine rabbit Antibody Thermo Fisher Scientific #BS-1278R Human, Mouse, Rat Rabbit 1/100 Saint Aubin, France
rabbit anti Cytokeratin 14 Abcam #ab181595 Human, Mouse, Rat Rabbit 1/500 Cambridge, United Kingdom
rabbit anti-IBA1 Wako #019-19741 Human, Mouse, Rat Rabbit 1/400 Richmond, VA, USA
mouse monoclonal anti-MR 6G1 Merck #MABS496 Human, Mouse, Rat Mouse 1/1500 Darmstadt, Germany
rabbit anti-GR Santa Cruz Biotechnology #sc-393232 Human, Mouse, Rat Rabbit 1/1500 Texas, USA
mouse anti-11β-HSD1(D-5) Santa Cruz Biotechnology #sc-518168 Human, Mouse, Rat Mouse 1/1500 Texas, USA
mouse anti-11β-HSD2(D-9) Santa Cruz Biotechnology #sc-365529 Human, Mouse, Rat Mouse 1/1500 Texas, USA
rabbit anti-4-Hydroxynonenal (4-HNE) Abcam #ab46545 Human, Mouse, Rat Rabbit 1/200 Cambridge, UK
rabbit anti-S100A9 Abcam #ab63818 Human, Rat Rabbit 1/300 Cambridge, UK
Secondary antibodies:
Alexa Fluor 488-conjugated goat anti-rabbit IgG Thermo Fisher Scientific #A11008 1/200 Saint Aubin, France
Alexa Fluor 488-conjugated donkey anti-mouse IgG Thermo Fisher Scientific #A21202 1/200 Saint Aubin, France
Alexa Fluor 594-conjugated goat anti-rabbit IgG Thermo Fisher Scientific #A11012 1/200 Saint Aubin, France
Alexa Fluor 594-conjugated donkey anti-mouse IgG Thermo Fisher Scientific #21203 1/200 Saint Aubin, France
biotinylated goat anti-mouse secondary antibody Vector Laboratories #BA-2000 1/500 Eurobio Scientific, Les Ulis, France
biotinylated goat anti-rabbit secondary antibody Vector Laboratories #BA-1000 1/500 Eurobio Scientific, Les Ulis, France

Validation

All primary antibodies used in this study were validated for the appropriate species and applications as stated on the manufacturers' websites. In addition, each antibody was independently validated in our laboratory for the intended experimental conditions. Validation included the use of appropriate negative controls, which showed no specific signal. Representative images of these negative controls and additional validation data are provided in the Supplementary Information.

Animals and other research organisms

Policy information about studies involving animals; ARRIVE guidelines recommended for reporting animal research, and Sex and Gender in Research

Laboratory animals

The study was conducted using female Sprague-Dawley rats (aged 12 weeks, 450-500 g, Janvier Labs, Le Genest-Saint-Isle, France).

Wild animals

The animals used in this study were transgenic rats overexpressing the human mineralocorticoid receptor (hMR) and control littlemates. These animals were bred in a controlled laboratory environment and were not wild-caught or field-collected. All experiments were performed under standard laboratory conditions following institutional guidelines for the care and use of laboratory animals. Adult female Sprague-Dawley rats (12 weeks, 450-500 g, Janvier Labs, Le Genest-Saint-Isle, France) were used

and kept in pathogen-free conditions with free access to food and water and housed in temperature-controlled room with a 12-h light/12-h dark cycle. Anesthesia was induced by intraperitoneal ketamine 100 mg/kg and Xylazine 10 mg/kg. Animal were euthanized by intraperitoneal injection of fatal dose of Euthasol® Vet.

Reporting on sex

The findings reported in this study apply exclusively to female animals, as only female subjects were used throughout the experiments.

Female animals were selected for this study based on evidence that females exhibit stronger inflammatory and stress responses, as documented in previous research1. Additionally, the smaller size of female animals compared to males results in more pronounced damage in the stress and inflammation models used, aligning with the goals of this study2.

Sex was explicitly considered in the study design, and only female animals were included to ensure a robust model of inflammation and stress. This appraoch was informed by prior evidence indicating stronger immune and stress responses in females compares to males.

Animals sex was verified during all this study. Verification was conducted through physical examination of external genitalia, following standard protocols for sex determination in laboratory animals.

As only female animals were included in this study, data were not disaggregated by sex. The findings reported here are specific to female subjects.

1. Klein, S. L & Flanagan, K. L. Sex differences in immune responses. Nat. Rev. Immunol. 16,626-638 (2016).
2. Fonkoue, I. T. et al. Sex differenes in Black Veterans with PTSD: women versus men have higher sympathetic activity, inflammation, and blunted cardiovagal baroreflex sensitivity. Clin. Auton. Res. Off. J. Clin. Auton. Res. Soc. 33, 757-766 (2023).

Field-collected samples

The animals used in this study were transgenic rats overexpressing the human mineralocorticoid receptor (hMR) and control littlemates. These animals were bred in a controlled laboratory environment and were not wild-caught or field-collected. All experiments were performed under standard laboratory conditions following institutional guidelines for the care and use of laboratory animals. Adult female Sprague-Dawley rats (12 weeks, 450-500 g, Janvier Labs, Le Genest-Saint-Isle, France) were used and kept in pathogen-free conditions with free access to food and water and housed in temperature-controlled room with a 12-h light/12-h dark cycle. Anesthesia was induced by intraperitoneal ketamine 100 mg/kg and Xylazine 10 mg/kg. Animal were euthanized by intraperitoneal injection of fatal dose of Euthasol® Vet.

Ethics oversight

All experiments were performed in accordance with the European Communities Council Directive 86/609/EEC and French national regulations. The experimental protocols were approved by local ethics committee (# 23478-2020010317557546 v4, Ethics committee n°005).

Note that full information on the approval of the study protocol must also be provided in the manuscript.

Clinical data

Policy information about clinical studies
All manuscripts should comply with the ICMJE guidelines for publication of clinical research and a completed CONSORT checklist must be included with all submissions.

Clinical trial registration

Provide the trial registration number from ClinicalTrials.gov or an equivalent agency.

Study protocol

Note where the full trial protocol can be accessed OR if not available, explain why.

Data collection

Describe the settings and locales of data collection, noting the time periods of recruitment and data collection.

Outcomes

Describe how you pre-defined primary and secondary outcome measures and how you assessed these measures.

Plants

Seed stocks

Report on the source of all seed stocks or other plant material used. If applicable, state the seed stock centre and catalogue number. If plant specimens were collected from the field, describe the collection location, date and sampling procedures.

Novel plant genotypes

Describe the methods by which all novel plant genotypes were produced. This includes those generated by transgenic approaches, gene editing, chemical/radiation-based mutagenesis and hybridization. For transgenic lines, describe the transformation method, the number of independent lines analyzed and the generation upon which experiments were performed. For gene-edited lines, describe the editor used, the endogenous sequence targeted for editing, the targeting guide RNA sequence (if applicable) and how the editor was applied.

Authentication

Describe any authentication procedures for each seed stock used or novel genotype generated. Describe any experiments used to assess the effect of a mutation and, where applicable, how potential secondary effects (e.g. second site T-DNA insertions, mosiacism, off-target gene editing) were examined.
