# Supplementary material for: Cross-species transcriptomics identify mineralocorticoid receptor pathway overactivation as a central driver of ocular rosacea
Source: Nat Commun. 2026 Apr 16;17:5247. doi: 10.1038/s41467-026-71945-4 (PMC13260418; doi:10.1038/s41467-026-71945-4)
Supplement: Supplementary file 2 — Peer Review File [file 41467_2026_71945_MOESM2_ESM.pdf]

nature portfolio

Peer Review File

Cross-species transcriptomics identify mineralocorticoid receptor pathway overactivation as a central driver of ocular rosacea

Corresponding Author: Professor Francine Behar-Cohen

This file contains all reviewer reports in order by version, followed by all author rebuttals in order by version.

A version of this paper was originally rejected for publication by Nature Communications, however that decision was reconsidered after appeal by the authors.

Version 1:

Reviewer comments:

Reviewer #1

(Remarks to the Author)
This is a well-edited manuscript on the role of overactive Mineralocorticoid Receptor (MR) activity in Ocular Rosacea (OC). A substantial number of humans suffer from OC that is associated with meibomian gland dysfuncton(MGD). This is exacerbated by aging and UVB exposure. The overactive MR is central to MGD and to the symptoms of OC. The authors demonstrate with the use of a transgenic rat that expresses the overactive human MR that, following UVB-exposure, expresses the genes and pathology associated with OC in humans. Not only does this model provide insight into the genes associated with OC (and skin rosacea), but it also reveals an overlap with both conditions and MGD, specifically in S100A9 expression. The manuscript shows S100A9 overexpression in human lid tissues with OC, and that it is overexpressed in the transgenic mice. In addition, the overactive MR impacts MG stem cells, impairing renewal that further exacerbates the disease. The conclusions are supported by the results, making a significant advancement that provides new information and questions for further study into the role of MR in MGD and treatment for OC.

Reviewer #3

(Remarks to the Author)
In this manuscript, Zhu et al show increased mineralocorticoid receptor expression in a limited number of rosacea specimens compared to age and gender matched controls, and use a rat model of mineralocorticoid receptor overexpression to demonstrate changes in eyelid expression that correlate with reduced Meibomian gland proliferation and increased fibrosis that become more apparent with aging. The authors went on to show that MR overexpression worsened UV-induced skin irritation and corneal surface damage. These findings have potential clinical translational significance and offer insights into a novel potential mechanism of Meibomian gland dysfunction. Despite these strengths, there are many weaknesses that diminish the potential impact in its current form.
1) A main concern is the global overexpression of the MR, which leads to alteration in many organs as described in ref 68 of the text. Could these changes be secondary to systemic inflammation due to renal and cardiovascular deficiencies? In the absence of a suitable epidermis-specific expression in rats, the authors should at least determine whether the effects seen are dependent on eyelid expression and activity of this receptor by applying an MR antagonist, as they used previously in other contexts (fig s6). Does the antagonist prevent the overexpression-induced changes?
2) Rat model: Please describe if n=4 eyes come from one eye from four independent rats, or both eyes from two rats. Arguably, both eyes from the same animal are not completely independent, and thus would render the statistics for two independent animals unreliable.
3) Were the changes in 11bHSD1 expression in human tissues significantly increased? No quantification or statistics are provided for this staining.
4) Some of the authors' conclusions rely on extrapolations and should be further tested:
a. The authors say that the described changes in 11bHSD1 and D2 indicate local increased availability of cortisol. This should be measured. Is cortisol increased in the eyelids of subjects with rosacea? Otherwise, this remains pure speculation.
b. Similarly, the authors infer mitochondrial dysfunction from alterations in Tim23 and Tom20 expression. Actual

mitochondrial function should be assessed.
c. Changes in a handful of lipid metabolism genes does not confirm meibomian gland dysfunction as stated inline 151.
d. Also, lines 161-163 are an overstatement. There is no proof here that MGD causes the transcriptional changes, nor that these are specific to rosacea (e.g., upregulation of Tlr3, Clu, and CCL2 is very common in many inflammatory conditions. In fact, later on the authors state that "Notably, S100a9/S100A9 was the only gene both MR-specific in the rat OR model and associated with human MGD and OR pathology". Is the list of genes in page 6 also found in OR, or only S100a9?
5) GSEA analysis is not appropriate for very small sets of genes (e.g., 14 upregulated in fig 2). Normalization in this context is not accurate and 2-3 genes can lead to significant enrichment. This section should be rewritten to discuss involvement of specific genes, rather than GSEA signatures.
6) A role for S100a9 should be formally tested and not simply inferred by upregulatin of the gene. Does a neutralizing antibody prevent or revert the changes induced by MR overexpression?
7) The paragraph starting on line 501 discusses many gene expression changes forming "an interaction network involved in chemotaxis, IL-17, and Toll-like receptor signaling". In fact, those appear to be all NF-kB targets. All TLR2, TLR4, NF-kB, and MyD88 have been associated previously with rosacea, many of which assayed from ectropion specimens as described for fig 1 here. The findings here should be discussed in the context of the prior literature.

Minor:
Human subjects: Please describe how the diagnosis of rosacea was determined for the eight ectropion subjects.
Fig 6D: Fold change versus what?

Version 2:

Reviewer comments:

Reviewer #3

(Remarks to the Author)
The authors successfully addressed all prior concerns

Open Access This Peer Review File is licensed under a Creative Commons Attribution 4.0 International License, which permits use, sharing, adaptation, distribution and reproduction in any medium or format, as long as you give appropriate credit to the original author(s) and the source, provide a link to the Creative Commons license, and indicate if changes were made.
In cases where reviewers are anonymous, credit should be given to 'Anonymous Referee' and the source.
The images or other third party material in this Peer Review File are included in the article's Creative Commons license, unless indicated otherwise in a credit line to the material. If material is not included in the article's Creative Commons license and your intended use is not permitted by statutory regulation or exceeds the permitted use, you will need to obtain permission directly from the copyright holder.
To view a copy of this license, visit https://creativecommons.org/licenses/by/4.0/

Answers to reviews' questions

Reviewer #1 (Remarks for the Author):
This is a well-edited manuscript on the role of overactive Mineralocorticoid Receptor (MR) activity in Ocular Rosacea (OC). A substantial number of humans suffer from OC that is associated with meibomian gland dysfunction(MGD). This is exacerbated by aging and UVB exposure. The overactive MR is central to MGD and to the symptoms of OC. The authors demonstrate with the use of a transgenic rat that expresses the overactive human MR that, following UVB-exposure, expresses the genes and pathology associated with OC in humans. Not only does this model provide insight into the genes associated with OC (and skin rosacea), but it also reveals an overlap with both conditions and MGD, specifically in S100A9 expression. The manuscript shows S100A9 overexpression in human lid tissues with OC, and that it is overexpressed in the transgenic mice. In addition, the overactive MR impacts MG stem cells, impairing renewal that further exacerbates the disease. The conclusions are supported by the results, making a significant advancement that provides new information and questions for further study into the role of MR in MGD and treatment for OC.
We would like to thank the reviewer for the positive assessment of our manuscript and our work.

Reviewer #3 (Remarks for the Author):

In this manuscript, Zhu et al show increased mineralocorticoid receptor expression in a limited number of rosacea specimens compared to age and gender matched controls, and use a rat model of mineralocorticoid receptor overexpression to demonstrate changes in eyelid expression that correlate with reduced Meibomian gland proliferation and increased fibrosis that become more apparent with aging. The authors went on to show that MR overexpression worsened UV-induced skin irritation and corneal surface damage. These findings have potential clinical translational significance and offer insights into a novel potential mechanism of Meibomian gland dysfunction. Despite these strengths, there are many weaknesses that diminish the potential impact in its current form.
We would like to thank the reviewer for his favourable assessment of our scientific approach and our work, and we hope that the additional information we have provided will reinforce the points he raised.

Major concerns
1) A main concern is the global overexpression of the MR, which leads to alteration in many organs as described in ref 68 of the text. Could these changes be secondary to systemic

inflammation due to renal and cardiovascular deficiencies? In the absence of a suitable epidermis-specific expression in rats, the authors should at least determine whether the effects seen are dependent on eyelid expression and activity of this receptor by applying an MR antagonist, as they used previously in other contexts (fig s6). Does the antagonist prevent the overexpression-induced changes?

We thank the reviewer for this comment which has allowed to better explain our experimental strategy.

Transcription of hMR is directed by two alternative promoters, P1 and P2, which correspond to the 5'-flanking regions of the untranslated exons 1alpha and 1beta of the hMR gene, respectively. The P1 promoter directs transgene expression in all MR-expressing tissues, although the strength of its transcriptional activity varies among organs[1]. This is reason why we chose the P1 promoter for the generation of the P1.hMR rat model and demonstrate herein that the P1 promoter drives the hMR expression in the meibomian glands in both male and female (Figure 2) as well as in the cornea and conjunctiva of both male and female rats, without affecting the endogenous rat MR and GR expression (Supplementary figure 1 and 2). Yet, no clinically significant dysfunctions or deficiencies developed spontaneously in these tissues (Figure 2c and 2d) (and Figure S4) at age of 6 months, demonstrating that hyperactivation of the MR pathway predisposes to a pathological state, triggered by additional factors.

Under basal conditions, we also verified that up to one year of age, the rats did not show any kidney failure or dysfunction as analysed in a metabolic cage (see below) :

		WT		Tg1		
		Mean	SEM	Mean	SEM	p-value
URINE	Urine volume (ml)	8,17	0,59	8,67	1,75	0,98
	Urine Creatinine (mM)	28,33	2,57	30,04	3,27	0,75
	U Na/ U Créat (mM/mM)	6,59	1,52	6,43	1,43	0,90
	U K/ U Créat (mM/mM)	11,74	1,80	11,54	1,46	0,90
	U Cl/ U Créat (mM/mM)	6,44	1,50	6,03	1,56	0,76
	U Ca/ U Créat (mM/mM)	0,02	0,10	0,02	0,12	0,87
	U Mg/ U Créat (mM/mM)	0,12	0,45	0,09	0,42	0,80
	Na/K	0,57	0,37	0,55	0,37	0,80

N=6 per group

Figure. Metabolic results in urine for WT and P1.hMR rats

In addition, in the heart, we could not measure any significant change in the expression of genes, known to be deregulated in case of MR-induced heart disease, including col3a1 (cardiac fibrosis), Gal-3 (fibrosis, heart failure), catalase (oxidative stress), IL-6 (endothelial dysfunction, atherosclerosis, myocardial fibrosis) and BNP although hMR was expressed.

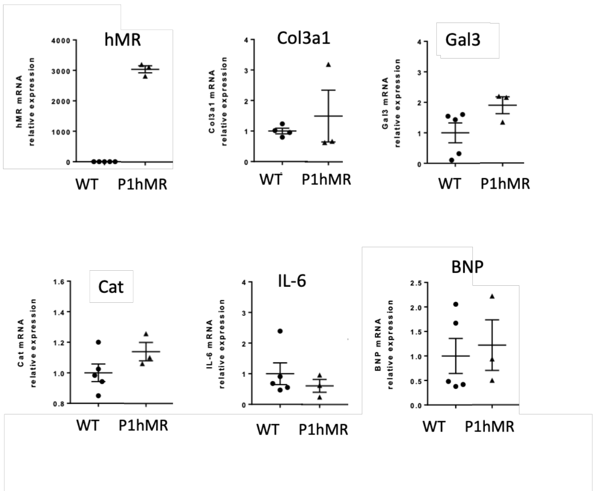

Altogether, these results demonstrate that the P1.hMR rat does not develop any spontaneous cardiac or kidney dysfunction under physiological conditions.

The ocular changes observed in the P1hMR rat with aging or after UV exposure is thus very unlikely to be secondary to a multi-organ dysfunction although it cannot be excluded that subtle MR-induced systemic inflammation could occur and contribute to the MGD pathogenesis.

Although a conditional transgenic model overexpressing MR only in the MG could be interesting to study, it might not be representative of a human clinical condition, where MR overactivation could be generalized and linked with low grade inflammation, without causing systemic diseases. We have shown increased expression of TLR-4 expression in conjunctival epithelium and peripheral blood mononuclear cells of patients with OR supporting the assumption that both systemic and local inflammation contribute to OR [Mol Vis 2024 Mar 30:30:211-218. eCollection 2024].

The local antagonism of MR by spironolactone eyedrops in the UV-induced rosacea model in P1hMR, was unfortunately not feasible. Indeed, UVB induces significant eyelid edema, which makes frequent eyedrop administration impractical and severely impairs retention of the drops on the ocular surface. Under such conditions, sustained therapeutic concentration of spironolactone cannot be achieved in the meibomian glands. Systemic treatment was not initially performed because we have conducted a clinical study in which patients with oculo-cutaneous rosacea had been treated with oral spironolactone for 3 months.

Here is a summary of the study:

We included 33 eyes of 33 patients diagnosed with oculo-cutaneous rosacea, with a mean age of 54.93 years. Among the patients, 21 were women and 12 were men. Tear break-up time (TBUT), meibography grade (meiboscore) were evaluated before treatment and three months after oral spironolactone (100 mg/day) indicated by the dermatologist. The study adhered to the principles of the Declaration of Helsinki and was approved by the Ethics Committee of Ankara Bilkent City Hospital (Approval No: E2-21-1656).

The results show improvement in TBUT at both the 1st and 3rd months after treatment compared with baseline ($p<0.05$). The meiboscore showed significant improvement only at the 3rd month after treatment ($p<0.05$).

	**Before Treatment** (Mean+SD)	**1M After Treatment** (Mean+SD)	**3M After Treatment** (Mean+SD)	**p**	**p***	**p****
**TBUT**	4.27±2.98	7.18±5.47	9.94±4.89	**0.011**	**<0.001**	**0.011**
**Meiboscore**	2.42±1.09	2.30±1.01	2.06±0.99	0.160	**0.002**	**0.009**

Table: Comparison of clinical parameters before and after treatment and controls
p value: Comparisons between before treatment and 1month after treatment
p*: Comparisons between before and after 3 month of treatment
p**: Comparisons between 1month and 3 month of treatment

Evaluation methods:
-TBUT was measured after instilling 5 µL of fluorescein into the conjunctival sac. After several blinks, the interval between the last blink and the appearance of the first dry spot on the corneal surface was recorded.
-Meibography grade (meiboscore) was calculated using the automated analyzer of the meibography device. (Sirius, CSO, Florence, Italy)

The complete results of this pilot study will be published separately. But it justified that we did not treat orally the rat as the clinical study is more valuable. However, as it does not prove that MGD can be alleviate by local administration of MR antagonists, we are currently preparing a clinical study to evaluate spironolactone drops in Human to test this hypothesis

In the conclusion of the present paper, we have mentioned that we are preparing a clinical study using spironolactone drops for LSCD secondary to OR.

Several additions were made in the paper as follows:

In material and methods:
L. 594-599: The human MR (hMR) gene is expressed under the control of the proximal P1 promoter of the NR3C2 as described previously (69,70). The P1 promoter drives the expression of hMR in in all MR-expressing tissues, although the strength of its transcriptional activity varies among organs(71). Using metabolic cages, we verified that up to one year of age, the P1.hMR rats did not develop kidney failure and no sign of MR-induced cardiac dysfunction was detected at the molecular level in the heart.

In the discussion, L. 471-476:

Since P1.hMR rats express hMR in all MR-expressing organs, it cannot be excluded that the initiation of MGD could result from MR overactivation both locally and systemically, although no signs of heart of kidney dysfunction was detected in these animals under basal conditions. The expression of MR in innate immune cells could also contribute to the pathogenesis of the disease in the rat model and in humans. Although a transgenic animal model in which hMR expression was limited to the meibomian glands would have been interesting, it would not have more accurately mimicked the human disease, which is known to be multifactorial and involve systemic immune and inflammatory factors.

In the limitations section: L.553

In the UV-induced OR model developed in P1.hMR, it was not feasible to test spironolactone drops as eyelid edema prevented the proper delivery of the drug and therefore whether local MR antagonism could alleviate MGD and OR will be evaluate in further clinical studies.

2) Rat model: Please describe if n=4 eyes come from one eye from four independent rats, or both eyes from two rats. Arguably, both eyes from the same animal are not completely independent and thus would render the statistics for two independent animals unreliable.

Thank you for this valuable comment.

In all experiments and main text of the manuscript, the rat number was considered as the experimental unit (highlighted in green all over the manuscript). Although both upper eyelids of each rat were exposed to UVB, only one eyelid per animal was processed for immunofluorescence, histology, or transcriptomic analysis, and therefore no paired-eye data were used for these outcomes.

Especially, for the statistical analysis of corneal fluorescein staining to evaluate corneal defects subsequent to UVB-induced MGD and OR, we added the details on L.629 :

"The final score was obtained by summing the scores of all four quadrants, and the scores from both eyes were averaged to obtain one value per animal for statistical analysis. Comparison was conducted between WT and P1.hMR at the timepoint 2weeks post-UVB (n = 6 rats)."

3) Were the changes in 11bHSD1 expression in human tissues significantly increased? No quantification or statistics are provided for this staining.

We have now performed quantitative analysis of 11β-HSD1 and 11β-HSD2 immunostaining in human MG tissues.

A modest but statistically significant increase in 11β-HSD1 expression, together with a decrease in 11β-HSD2 expression was measured. The quantitative data and corresponding statistical analyses have now been included in the revised manuscript L.118 and in Figure 1b.

4) Some of the authors' conclusions rely on extrapolations and should be further tested:
a. The authors say that the described changes in 11bHSD1 and D2 indicate local increased availability of cortisol. This should be measured. Is cortisol increased in the eyelids of subjects with rosacea? Otherwise, this remains pure speculation.

Tissue expression of 11β-HSD1 is a reliable and recognized marker of the conversion of cortisone to cortisol locally, but it would be necessary to develop an in-situ measurement of the cortisol/cortisone ratio. We are currently working collaboratively on the application of targeted mass spectrometry on tissue sections to better understand the local steroidome. But this technique is yet under development.

We have revised the manuscript to clarify that the expression of HSD1 does not constitute evidence of local (in situ) cortisol increase, as follow:

In the result section, L.101:

In the eyelid of OR samples, 11βHSD1 immunostaining was enhanced, while 11βHSD2 immunostaining was reduced (Fig. 1b), suggesting a potential local deregulation of cortisol/ cortisone ratio.

In the discussion: L.447, we have added:

The exact mechanisms of MR activation remain to be determined. It could be related to the overexpression of the receptor, abnormal local metabolism of corticoids and / or to ligand-independent MR activation. Whether cortisol is increased locally in the MG and eyelids cannot be extrapolated solely by the 11βHSD activity and should be further explored by in situ steroidome analysis.

b. Similarly, the authors infer mitochondrial dysfunction from alterations in Tim23 and Tom20 expression. Actual mitochondrial function should be assessed.

Among the pathogenic mechanisms favoured by MR overactivation in P1.hMR rats, we mentioned mitochondrial dysfunction base on:

- Genes involved in oxidative phosphorylation that were deregulated in MG of P1.hMR rats.
- Oxidative stress markers, including 4-HNE and 3-NT (Fig. 4d and 4e), enhanced in P1.hMR rats. Both 4-HNE and 3-NT are well-established markers of mitochondrial oxidative injury, leading to impaired mitochondrial bioenergetics.

To support this finding, we now include immunostaining for acrolein (Fig. 4f), a highly reactive aldehyde generated during lipid peroxidation in rats exposed to UVB. Although not specific to mitochondria, acrolein is a marker of mitochondria-driven oxidative injury. The punctate

staining suggesting mitochondrial staining (white arrows)(Figure below) is significantly increased in P1h.MR MG.

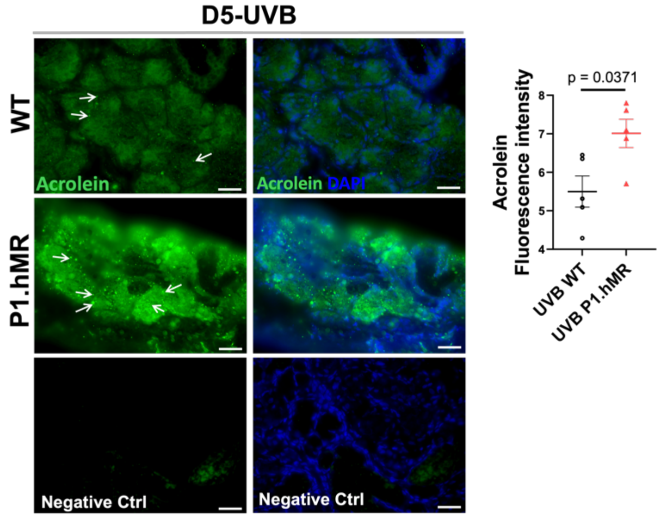

Figure. Acrolein staining (green color) in MG tissues on day 5 of UVB was determined by immunohistochemistry and quantified by ImageJ based on staining intensity (n = 5 rats). Data expressed as mean ± SEM. ns, $p > 0.05$ (Mann-Whitney U test).

Additional IHC for 8-hydroxydeoxyguanosine (8OHdG), a marker for oxidative DNA damage was performed. It was significantly more pronounced in both the nuclei and mitochondria of meibocytes in P1.hMR rats compared with wild-type rats exposed to UVB, suggesting that MR overexpression enhances the susceptibility of meibocytes to oxidative stress, particularly mitochondrial DNA damage and dysfunction (Figure below).

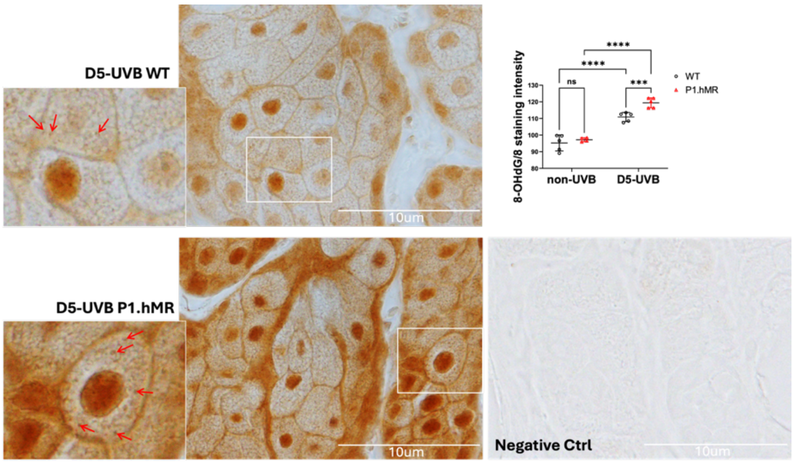

Figure. 8-OHdG/8 protein expression in MG tissues on day 5 of UVB was determined by immunohistochemistry and quantified by ImageJ based on staining intensity (n = 4-5 rats). Data expressed as mean ± SEM. Ns, $p > 0.05$ (Two-way ANOVA with Sidak's multiple comparison).

We have changed the sentence in the results section as follows, L.143:

In addition, a reduced expression of two mitochondrial proteins, TOM20 and TIM23 (Fig. 2f), could suggest an alteration of mitochondria metabolism , which is supported by deregulation

of the oxidative phosphorylation pathway in the transcriptomic analysis of MG from transgenic rats.

In addition, we have added the following results in the result section and in FIG4f and g, L. 241:
Enhanced expression of acrolein, a non-specific marker of mitochondria-driven oxidative injury, further supported enhanced lipid peroxidation in MG of P1.hMR rats (Fig. 4f). In addition, 8OHdG, a marker for oxidative DNA damages was significantly more pronounced in both the nuclei and mitochondria of meibocytes in P1.hMR rats compared with WT, suggesting that MR overexpression enhances the susceptibility of meibocytes to oxidative stress, particularly mitochondrial DNA damage and dysfunction (Fig. 4g).

Because of the extremely small tissue mass, mitochondria could not be isolated from rat meibomian glands, rendering direct evaluation of mitochondrial function impossible.

c. Changes in a handful of lipid metabolism genes does not confirm meibomian gland dysfunction as stated inline 151.
We fully agree and this is why at 6 months of age we stated that (L. 139) "no significant MG dropout and no histological alterations were observed in the in 6 -months-old transgenic rats«

No significant consequences on MG lipid synthesis and no signs of hyperkeratinization were observed in transgenic rats (Fig. S2a and S2b).

Although a decline in MG stem cells and mitochondrial metabolism was observed in P1.hMR rats, suggesting MGD initiation, no signs of OR was observed in 6-month old rats » (L.156).

We have modified the description that was mentioned on line 156 as follows:

The deregulated genes identified in in the RNA seq analysis and validated by RT-PCT in separate samples (look in supplementary Figure S3), have limited clinical consequence, at that stage, on the phenotype. Nevertheless, these genes were also found to be deregulated in human MGD tissues and are associated with biologically relevant functions related to the disease (L.166)

d. Also, lines 161-163 are an overstatement. There is no proof here that MGD causes the transcriptional changes, nor that these are specific to rosacea (e.g., upregulation of Tlr3, Clu, and CCL2 is very common in many inflammatory conditions. In fact, later on the authors state that "Notably, S100a9/S100A9 was the only gene both MR-specific in the rat OR model and associated with human MGD and OR pathology". Is the list of genes in page 6 also found in OR, or only S100a9?

In L.161 to 163, we listed the genes identified by the RNA seq that were further validated by RT-PCR in additional rat samples. We found it worth to note that such genes and related pathways were also found to be deregulated in human MGD and in the skin rosacea[2–4].

We fully agree with the reviewer and wrote that such genes are not specific and not causative for MGD as they have also been found in skin rosacea. Their eventual role in disease pathogenesis is unknown.

To strengthen this idea, we have now added the following sentence, L. 164 :

"These genes and related pathways are not specific to MGD and whether such deregulated genes play a role in MGD has not been studied but interestingly they were also deregulated in human tissues with MGD and skin rosacea."

e. Is the list of genes in page 6 also found in OR, or only S100a9?

The list of genes on P.6 are the genes that were deregulated in the MG of P1hMR at 6 month of age, at a time when there was no clinical phenotype but rather a moderate change in the MG on histology and immunohistochemistry. They are thus genes, induced by the deregulation of MR at an early stage, when no clinical consequence of MGD is noticed.

It was interesting to note that those genes or related pathways were also identified in MGD patients or animal studies[2,5,6] by other teams and in the skin of rosacea patients[4,7].

But in human samples, they could be a consequence of multiple causative factors. In the rat, they are a consequence of MR overactivation that may potentially related to the MGD and OR pathogenesis.

5) GSEA analysis is not appropriate for very small sets of genes (e.g., 14 upregulated in fig 2). Normalization in this context is not accurate, and 2-3 genes can lead to significant enrichment. This section should be rewritten to discuss involvement of specific genes, rather than GSEA signatures.

As mentioned in the results section, L. 166 and in the supplementary figure 4, we validated by quantitative PCR the differentially expressed genes and we compared them to known data in humans with MGD and OR. Supplementary Figure 4 shows in a table the list of genes deregulated in P1hMR that have been validated by RT-PCR and classes them according to their known functions in MGD according to the literature (ref 30, 31, 32). These genes have thus been described specifically.

Nevertheless, the GSEA analysis remains informative and valid in this case for the following reasons:

-GSEA (Gene Set Enrichment Analysis) was designed to extract biological signals rather than to focus on individual genes reaching classical significance thresholds, which is one of the main reasons the method was developed[8].

- Several studies in transcriptomics emphasize that complex biological processes are frequently driven by small but coordinated expression changes across pathways, which different genes expression alone cannot capture. From a statistical point of view the analysis of groups instead of individual genes is advantageous as this typically increases power and reduces the dimensionality[9].

Notably, the GSEA performed here included all genes, not only those that were significantly regulated, but also genes whose expression changes did not reach conventional significance thresholds. Despite this, the coordinated alterations across these genes resulted in statistically significant enrichment of biofunctional pathways in the GSEA analysis. This provides valuable insight into the biofunctional changes induced by MR overexpression and the regulated pathways relevant to pathogenesis of MGD and OR.

6) A role for S100a9 should be formally tested and not simply inferred by upregulation of the gene. Does a neutralizing antibody prevent or revert the changes induced by MR overexpression?

We agree that the role of S100A9 cannot in any way be inferred from any of the experiments presented here. The aim of this work was not to demonstrate the role of S100A9. Our work has identified S100A9 as a candidate biomarker for MR activation in ocular rosacea. This finding was further confirmed by the fact that spironolactone decreased its expression (Figure S6d) in the cornea of LSCD rat model.

If confirmed by further studies, it could be used as a biomarker of MR activation, as rosacea is a multifactorial disease.

As stated L. 542 « As S100A9 is detectable in tears and correlates with MGD severity in dry eye disease (ref 65), it may serve as a non-invasive biomarker of MR activation in OR, offering a potential tool for patient stratification and MR-targeted therapies. »

The potential pathogenic role of S100A9 should be studied in transgenic animals with S100A9 invalidation and MR overexpression, for example. But here we would only present its role as a functional biomarker linked with MR overexpression and OR patients, which could help us select the patients responsive to MR antagonist treatment.

We have clarified this point in the discussion section as follows, L. 546

"S100A9 could contribute to MG damage and dysfunction. But the exact role of S100A9 should be further evaluated using specific deletion of S100A9 in MGD animal models." As well as in the abstract as follows (L40-44):

"we identified S100A9 as a specific downstream target and biomarker of MR activation. Local pharmacological MR antagonism suppressed S100A9 expression. These findings establish MR overactivation as a unifying pathogenic driver of MGD and OR and identify MR blockade as a promising therapeutic strategy, with S100A9 as a candidate biomarker for patient stratification and treatment monitoring."

It was also already stated L.546 as follows in the limitation section:

"However, the specific effects of the downstream target S100A9 have not yet been investigated."

7) The paragraph starting on line 501 discusses many gene expression changes forming "an interaction network involved in chemotaxis, IL-17, and Toll-like receptor signalling". In fact, those appear to be all NF-kB targets. All TLR2, TLR4, NF-kB, and MyD88 have been associated previously with rosacea, many of which assayed from ectropion specimens as described for fig 1 here. The findings here should be discussed in the context of the prior literature.

Indeed, the aim of our analysis was to cross-reference deregulated genes in our transgenic rat models with genes already known and published by others in MGD and OR. That is why we cite these already known genes and have cited the references of the studies that identified them (ref 36, 37, 58, 60, 61).

Minor concern:

1) Human subjects: Please describe how the diagnosis of rosacea was determined for the eight ectropion subjects.

Patients and control subjects were diagnosed by both an ophthalmologist and a dermatologist. OR was diagnosed based on ocular symptoms and clinical signs of OR including lid margin telangiectasia, blepharitis, blepharoconjunctivitis, and meibomian gland dysfunction, based on examination of the MG orifices, irregularity of the palpebral area, meibomian gland loss and abnormal vascularization of the eyelid margins. Inflammatory conditions other than OR were excluded. Resection surgeries were performed due to the diagnosis of ectropion, entropion, and other necessary conditions on the patients. The surgical pieces were immediately fixed in 10% formalin for 24 hours, dehydrated, embedded in paraffin wax and cut into 7 um thick slices and stained with hemalum-eosine, for histological analysis. All patients signed written consent for non-opposition to the use of surgical waste in research. Diagnosis of OR patients and control subjects were further confirmed by a pathologist on the sections. Additional sections were used for specific staining relative to the present study.

Accordingly we added the details in the revised manuscript on L.591:

Patients and control subjects were diagnosed by both an ophthalmologist and a dermatologist OR was diagnosed based on ocular symptoms and clinical signs of OR including lid margin telangiectasia, blepharitis, blepharoconjunctivitis, and meibomian gland dysfunction, based on examination of the MG orifices, irregularity of the palpebral margin, meibomian gland loss

and abnormal vascularization of the eyelid margins. Inflammatory conditions other than OR were excluded

And L.599:

Diagnosis of OR patients and control subjects were further confirmed by a pathologist on the sections.

2) Fig 6D: Fold change versus what?

We believe that the fold-change (FC) values referred here are those presented in Fig. 6b. This figure is intended to compare commonly regulated genes (previously reported to be relevant to MGD and OR pathogenesis) in WT versus P1.hMR rats caused by UVB-induced OR rat model. Notably, MR overexpression in P1.hMR rats resulted in more pronounced regulation of these genes, as reflected by higher FC values. Specifically, the FC values in the UVB-WT group represent gene regulation between UVB-exposed WT rats and non-UVB WT controls, whereas the FC values in the UVB-P1.hMR group represent gene regulation between UVB-exposed P1.hMR rats and non-UVB P1.hMR controls. These details have now been added to the figure legend, as highlighted at L. 375-76

Figure 6. Transcriptomic analyses of UVB-induced MGD in WT and P1.hMR rats.

a. Venn diagrams illustrating differentially expressed genes (DEGs) in the MG of WT and P1.hMR rats exposed to UVB. DEGs critically associated with MGD and rosacea are listed. b. Fold Change (FC) values of representative common DEGs in UVB-WT (UVB WT vs. non-UVB WT) and UVB-P1.hMR (UVB P1.hMR vs. non-UVB P1.hMR) rats. c. Enrichment analysis of common DEGs using GOBP database. d. Enrichment analysis of common DEGs using KEGG database. e. Enrichment analysis of DEGs exclusively identified in WT rats. f. Enrichment analysis of DEGs exclusively identified in P1.hMR rats. n = 3-5 rats in each group.
